# Amplification of microbial DNA from bacterial extracellular vesicles from human placenta

**DOI:** 10.3389/fmicb.2023.1213234

**Published:** 2023-07-13

**Authors:** Ramkumar Menon, Kamil Khanipov, Enkhtuya Radnaa, Esha Ganguly, Giovana Fernanda Cosi Bento, Rheanna Urrabaz-Garza, Ananth Kumar Kammala, Jerome Yaklic, Richard Pyles, George Golovko, Ourlad Alzeus G. Tantengco

**Affiliations:** ^1^Division of Basic Science and Translational Research, Department of Obstetrics and Gynecology, The University of Texas Medical Branch at Galveston, Galveston, TX, United States; ^2^Department of Pharmacology and Toxicology, The University of Texas Medical Branch at Galveston, Galveston, TX, United States; ^3^Department of Pediatrics, The University of Texas Medical Branch at Galveston, Galveston, TX, United States; ^4^Department of Physiology, College of Medicine, University of the Philippines Manila, Manila, Philippines; ^5^Department of Biology, College of Science, De La Salle University, Manila, Philippines

**Keywords:** bacterial outer membrane vesicles, EVS, metagenomics, parturition, placenta, pregnancy

## Abstract

**Introduction:**

The placenta is essential for fetal growth and survival and maintaining a successful pregnancy. The sterility of the placenta has been challenged recently; however, the presence of a placental microbiome has been controversial. We tested the hypothesis that the bacterial extracellular vesicles (BEVs) from Gram-negative bacteria as an alternate source of microbial DNA, regardless of the existence of a microbial community in the placenta.

**Methods:**

Placentae from the term, not in labor Cesareans deliveries, were used for this study, and placental specimens were sampled randomly from the fetal side. We developed a protocol for the isolation of BEVs from human tissues and this is the first study to isolate the BEVs from human tissue and characterize them.

**Results:**

The median size of BEVs was 130–140 nm, and the mean concentration was 1.8–5.5 × 10^10^ BEVs/g of the wet placenta. BEVs are spherical and contain LPS and ompA. Western blots further confirmed ompA but not human EVs markers ALIX confirming the purity of preparations. Taxonomic abundance profiles showed BEV sequence reads above the levels of the negative controls (all reagent controls). In contrast, the sequence reads in the same placenta were substantially low, indicating nothing beyond contamination (low biomass). Alpha-diversity showed the number of detected genera was significantly higher in the BEVs than placenta, suggesting BEVs as a likely source of microbial DNA. Beta-diversity further showed significant overlap in the microbiome between BEV and the placenta, confirming that BEVs in the placenta are likely a source of microbial DNA in the placenta. Uptake studies localized BEVs in maternal (decidual) and placental cells (cytotrophoblast), confirming their ability to enter these cells. Lastly, BEVs significantly increased inflammatory cytokine production in THP-1 macrophages in a high-dose group but not in the placental or decidual cells.

**Conclusion:**

We conclude that the BEVs are normal constituents during pregnancy and likely reach the placenta through hematogenous spread from maternal body sites that harbor microbiome. Their presence may result in a low-grade localized inflammation to prime an antigen response in the placenta; however, insufficient to cause a fetal inflammatory response and adverse pregnancy events. This study suggests that BEVs can confound placental microbiome studies, but their low biomass in the placenta is unlikely to have any immunologic impact.

## 1. Introduction

The human placenta is one of the organs vital for fetal growth, maintenance of pregnancy, and ensuring safe delivery of the fetus (Maltepe and Fisher, 2015). The placenta is the gatekeeper for transporting all essential materials across the feto-maternal interface. It also maintains the immune and endocrine balance necessary for the development and survival of the fetus (Hafez, 2017). Despite this understanding, this critical organ is understudied in many regards, including whether it supports a microbial community.

The human microbiome project (The NIH HMP Working Group et al., 2009; Human Microbiome Project C, 2012a) has opened up a new avenue of research relating to the placental functions that questioned the sterile nature of this organ (Human Microbiome Project C, 2012b). Recent technological advances have enhanced the ability to screen for microbiota in various human tissues, leading to the recognition of bacterial DNA at sterile sites (The NIH HMP Working Group et al., 2009). Reports of placental microbiome communities during normal gestation (Aagaard et al., 2014; Antony et al., 2015; Zheng et al., 2017; Parnell et al., 2017a; Chu et al., 2018; Zhu et al., 2018) have hypothesized that microbes and their metabolites contribute to placental physiology (Aagaard et al., 2014; Parris et al., 2021). Microbes have been isolated from the placenta in abundance from women with stillbirth, infection-associated preterm birth, and preterm prelabor rupture of the membrane (Rolschau, 1978; Doyle et al., 2014; de Goffau et al., 2019; Chopra et al., 2020). These microbes are considered intruders of the otherwise sterile placental tissue, causing pathologic and immunobiological changes that contribute to various adverse pregnancy outcomes (Mysorekar and Cao, 2014; Payne and Bayatibojakhi, 2014; de Goffau et al., 2019). Recent reports by many laboratories using an array of techniques, primarily culture-independent, nucleic acid-based approaches, have suggested the existence of a placental microbiome during normal pregnancy. However, the presence of the placental microbiome has been controversial as many investigators questioned various technical aspects of the approaches, including microbial contamination during placental sampling, nucleic acid extraction, and subsequent amplification that create noise rather than true detection of viable microbiota (Bushman, 2019; de Goffau et al., 2019; Theis et al., 2019, 2020b; Kuperman et al., 2020; Campisciano et al., 2021; Fricke and Ravel, 2021). A recent systematic review has suggested that the placental microbiome has low biomass, and current evidence is insufficient to predict a functional role for the communities (Zakis et al., 2022). Controversies surrounding the placental microbiome and the search for an alternate source of microbial constituents led us to hypothesize that bacterial extracellular vesicles (BEVs) are a potential source of microbial materials, including nucleic acid in the placenta. Additionally, isolation and amplification of nucleic acid released from or contained within the BEVs may create a molecular signature mistakenly assigned as the placental microbiome.

Bacterial extracellular vesicles are bi-layered structures and one of the secretory mechanisms used by Gram-negative bacterial cells (Kulp and Kuehn, 2010; Pathirana and Kaparakis-Liaskos, 2016; Roier et al., 2016). Like the extracellular vesicles from eukaryotic cells (EVs, microvesicles, and apoptotic bodies; Théry et al., 2018; Kalluri and LeBleu, 2020), prokaryotes also release endocytic BEVs to communicate and coordinate group behavior; a mechanism referred to as quorum sensing (Mashburn and Whiteley, 2005). Besides communication, BEVs also are involved in resistance to environmental stressors (McBroom and Kuehn, 2007; Jha et al., 2017), biofilm formation (Esoda and Kuehn, 2019; Knoke et al., 2020; Zhao et al., 2022), invasion of host tissue (Chen et al., 2017; Finethy et al., 2017), the determination of cell fate (Losier et al., 2019), and host immune response (Kaparakis-Liaskos and Ferrero, 2015; Finethy et al., 2017; Jha et al., 2017; Giordano et al., 2020; McMillan et al., 2021). BEVs also contribute to microbial genetic material transfer and aid the exchange of toxins and virulence factors between cells (Kulp and Kuehn, 2010). Recent studies have shown that Gram-positive bacteria also secrete BEVs (Brown et al., 2015; Briaud and Carroll, 2020). BEV cargo contains various microbial metabolites, toxins, virulence factors, and microbial nucleic acids protected by the lipid bilayer structure. Previous studies showed the presence of DNA and RNA from BEVs from different bacteria, such as *Escherichia coli*, *Pseudomonas aeruginosa*, and *Staphylococcus aureus* (Bitto et al., 2017, 2021; Aktar et al., 2021). Animal model studies have shown that Group B *Streptococcus*-derived BEVs that contain antigenic cargo can cause preterm birth suggesting that the live microbe is not required to cause intraamniotic inflammation. BEVs, therefore may represent a form of sterile inflammation (Surve et al., 2016).

Bacterial extracellular vesicles have been isolated from various human biological fluids. Based on data from 16S ribosomal RNA (16S rRNA) gene sequencing after amplifying the V1-V3 region of the 16S rDNA, Yoo et al. reported that urine samples from pregnant and nonpregnant women contained BEVs. The profile of detected sequences was distinct between pregnant and nonpregnant groups, suggesting that microbial community changes were reflected in BEVs detected in biological samples (Yoo et al., 2016). This data support our hypothesis that systemic spread of BEVs occurs, leading to deposition in distant tissues, including the placenta. The microbial communities in the human body each generate BEVs as part of the secretory function of selected community members. A portion of the BEVs would be carried hematogenously, where they would be delivered to blood-rich sites like the placenta. Although blood and gut microbiome BEVs are reported (Muraca et al., 2015), to our knowledge, no reports are currently available on physiologic and pathologic contributions of BEVs in other human tissues. No documented methods to isolate BEVs from human tissues are currently available.

We first developed a reproducible method to test our hypothesis to isolate BEVs from placenta tissues based on previous literature and our experiences with similar-sized particles (Tulkens et al., 2020; Shahin et al., 2021; Shepherd et al., 2021; Tantengco et al., 2021b). We then characterized BEVs for size, shape, cargo, and molecular markers. DNA isolated from BEVs was subjected to 16 s rRNA amplification and sequencing, followed by bioinformatics analysis. We report successful isolation and characterization of BEVs from term human placenta. The microbial DNA in the isolated BEV was assigned to specific taxa and was independent of any contaminants present in various reagents used to isolate BEVs or DNA detection. We also confirmed the immunogenicity of BEVs isolated from the placenta following exposure to human THP1 cells (macrophage cell line). Our study does not prove or disprove the presence of a placental microbiome. Still, it identifies an alternate source of microbial DNA in the human placenta that must be considered in microbiome studies.

## 2. Materials and methods

### 2.1. Placental collection

**Figure 1:**
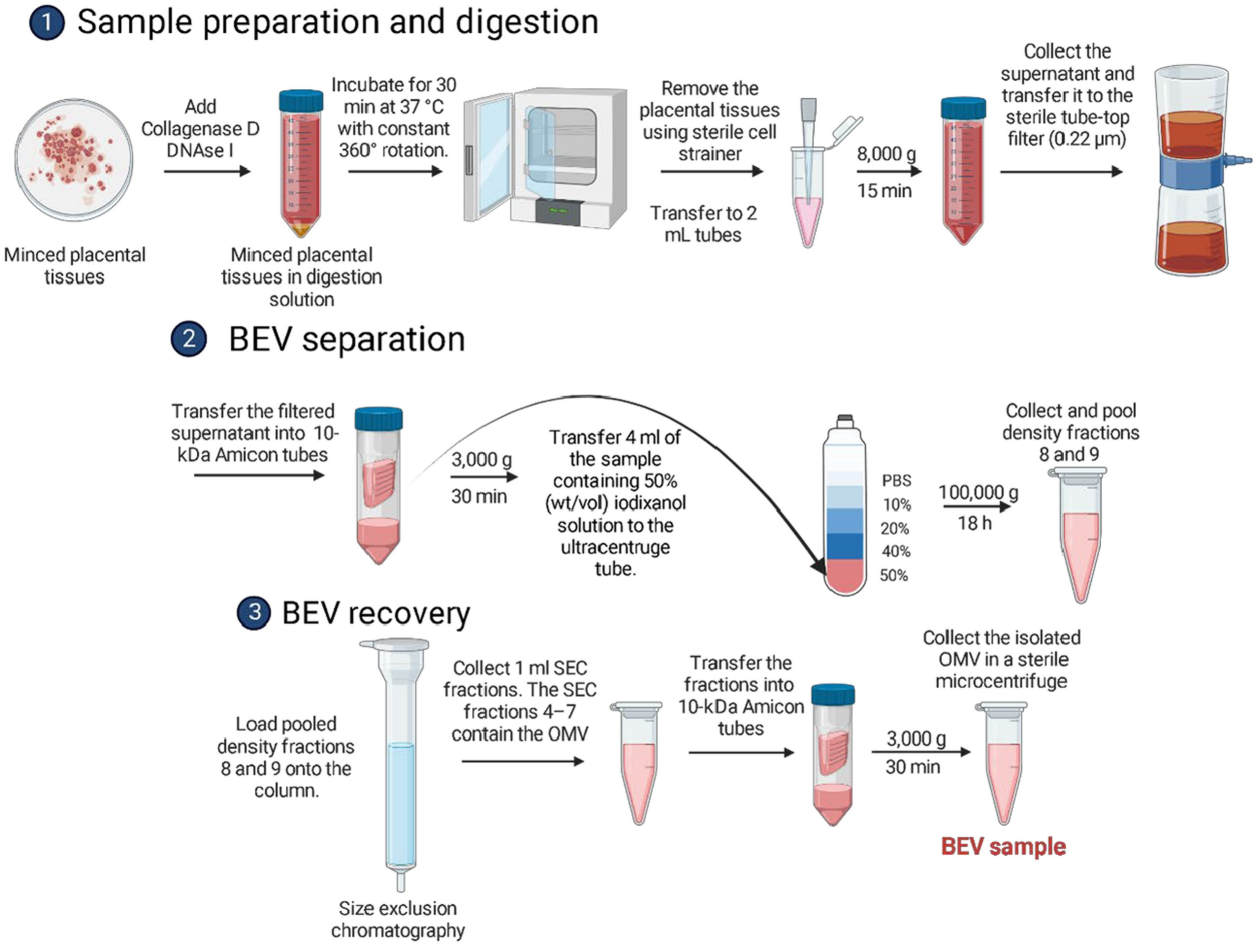
Schematic diagram showing the isolation of bacterial extracellular vesicles from human placental tissues using differential centrifugation, density-gradient ultracentrifugation, and size exclusion chromatography.

The BEV isolation protocol from placental tissues (Figure 1) was adapted from the previous protocol developed by Tulkens et al. (2020) with several modifications. This previous protocol demonstrated that BEVs can be isolated without other human-derived extracellular vesicle contamination. The placentas (*n* = 35) were collected from elective cesarean delivery patients. The placentas were placed in sterile containers using sterile gloves and instruments. Approximately, 3 × 3 cm cross-sectional tissue samples were collected from all four quadrants of the placenta, each at about 4 cm from the cord insertion site (Supplementary Figure S1). The fetal membrane was not included in the collected placental tissues. Our sampling was primarily from the villous area and avoided the chorionic and basal plate and the feto-maternal interface. This sampling approach prevented the accidental inclusion of microbes expected to be present on the maternal side or other maternal uterine tissues that may harbor microbes and confound our findings. Approximately 10 g of placental tissue was used for the BEV isolation protocol.

### 2.2. BEV isolation

#### 2.2.1. Crude extract preparation

The placental tissue was transferred in a sterile petri dish with 10 mL endotoxin-free PBS. The tissue was gently sliced using a sterile disposable scalpel until all pieces of the tissue had a homogenous size of 2 mm × 2 mm × 2 mm. The tissue was then transferred in a sterile 50 mL tube containing 15 mL endotoxin-free PBS with collagenase D (2 mg/mL) and DNAse I (40 U/mL). The tissue was incubated for 30 min at 37°C with constant 360° rotation. After digestion, the dissociated tissue was filtered using a 70 μm cell strainer. The filtered tissue sample was transferred into sterile 2 mL tubes and centrifuged at 8,000 × *g* for 15 min at 4°C. The supernatant was collected and transferred in sterile 2 mL tubes. The supernatant was then filtered using a 0.22 μm filter and moved into 10-kDa Amicon tubes and was centrifuged at 3,000 × *g* for at least 30–40 min at 4°C until the sample was concentrated to at least 667 μL. The sample was transferred in a 2 mL tube and was placed immediately on ice.

#### 2.2.2. BEV separation and manual density-gradient fraction collection

For BEV separation, a density gradient was prepared using iodixanol solutions. Iodixanol was reported to be non-ionic, non-toxic to cells and metabolically inert (Ford et al., 1994). The working solution was prepared by mixing one volume of gradient buffer A (Supplementary Methods) with Optiprep. The 10, 20, and 40% (weight/volume) iodixanol solutions were prepared by mixing the working solution with gradient buffer B (Supplementary Methods). The 50% iodixanol sample solution was prepared by mixing the 667 μL BEV sample with 3.33 mL of OptiPrep. The Optiprep is a 60% (wt/vol) solution of iodixanol in water. The sample solution was mixed by pipetting up and down until it became homogeneous. To prepare the density gradient ultracentrifugation tube, approximately 4 mL of the BEV crude extract sample containing 50% (wt/vol) iodixanol solution was carefully transferred to the bottom of the ultracentrifugation tube. The tube was then tilted to 70°, and 4 mL of the 40% (wt/vol) iodixanol solution was carefully transferred (drop by drop) to the surface of the liquid using a P1000 pipette. The same procedure was done by adding 4 mL of 20% (wt/vol) iodixanol solution, followed by 3.5 mL of the 10% (wt/vol) iodixanol solution. Finally, 1 mL of endotoxin-free PBS was added to the topmost surface of the ultracentrifugation tube. This was subjected to ultracentrifugation at 100,000 × *g* for 18 h at 4°C with an acceleration profile of 0 and a deceleration profile of 9 in an ultracentrifuge. After completion of the ultracentrifugation, the tube was carefully transferred on ice to a sterilized workspace where 1-mL fractions were carefully collected from the top of the ultracentrifugation tube and transferred to sterile sample tubes. The 8 and 9th fractions were pooled and placed immediately on ice. Based on initial studies, these fractions contained the BEVs from the sample.

#### 2.2.3. BEV recovery by size exclusion chromatography

The size exclusion chromatography (SEC) column was prepared using Sepharose beads (Supplementary Methods). The SEC column was washed with at least 10 mL of endotoxin-free PBS. The pooled 8th and 9th fractions (approximately 2 mL) were loaded onto the top of the SEC column. After loading, 1-mL SEC fractions were collected. The first 3 mL was void volume, while the SEC fractions 4–7 contained the pure BEVs. Fractions 4–7 were transferred into 10-kDa Amicon tubes to reduce the volume to 100 μL and increase the BEV concentration. The isolated BEVs were placed in sterile 2 mL tubes and stored in a − 80°C freezer until use.

### 2.3. BEV characterization

#### 2.3.1. Determination of live bacteria

To determine the sterility of the isolated BEV, approximately 20 μL of the isolated BEV samples was spread on LB-agar plates (Difco™ nutrient agar, BD Biosciences, San Jose, CA, United States, Cat. # BD213000, Lot. 9218604; in triplicates). Bacterial growth was checked every 24 h during incubation at 37°C until 72 h.

#### 2.3.2. Immunogold staining and transmission electron microscopy

The shape and size of BEVs and the expression of surface BEV markers were determined using immunogold staining and transmission electron microscopy (Jeol, Peabody, MA, United States). Briefly, 20 μL of BEV suspension in 1× PBS was applied onto formvar/carbon-coated 200-mesh copper grids and left to dry at room temperature for 10 min. The grids were incubated with mouse anti-LPS and rabbit anti-ompA at 1:1,000 in 1% bovine serum albumin (BSA)-PBS for 30 min at room temperature. The grids were then washed in 1% BSA-PBS and then incubated with goat anti-mouse IgG antibody (1:20 in 1% BSA-PBS) labeled with 6-nm gold particles and goat anti-rabbit IgG antibody (1:20 in 1% BSA-PBS) labeled with 15-nm gold particles (Electron Microscopy Sciences, Hatfield, PA, United States) for 30 min at room temperature. Finally, the grids were washed with deionized water, fixed in 2% aqueous glutaraldehyde for 10 min, and then rinsed with deionized water. The BEV-covered grids were negatively stained using 2% aqueous uranyl acetate for 1 min, filtered through a 0.2 μm filter, and left to dry at room temperature. The grid was directly placed into a grid box until observation. The grids were examined in a Philips CM-100 transmission electron microscope at 60 kV. Images were acquired on a bottom-mounted CCD camera Orius SC2001 (Gatan, Pleasanton, CA, United States). A minimum of five frames were viewed per sample.

#### 2.3.3. Nanoparticle tracking analysis with ZetaView

Nanoparticle tracking analysis was performed using the ZetaView PMX 110 (Particle Metrix, Meerbusch, Germany) and its corresponding software (ZetaView 8.05.05). Frozen placental BEVs were thawed on ice. A 1:50 dilution of the BEV sample was made with PBS. Samples were loaded in the ZetaView Nanoparticle Tracking Analyzer, and the number of particles/mL and the size distribution was quantified for each sample.

#### 2.3.4. Western blot analysis for EVs and BEV markers

The placental BEVs were lysed using 10× RIPA lysis buffer (0.50 M Tris pH 8.0, 1.50 M NaCl, 10% Triton X, 5% sodium deoxycholate, and 10% SDS) supplemented with protease and phosphatase inhibitor cocktail (1:10 v/v). The lysis mixture was vortexed for 30 s, sonicated for 30 s, and kept on ice for 30 min. Protein concentrations in the prepared samples were determined using a Pierce BCA protein assay kit (Pierce, Rockford, IL, United States). The protein samples (*n* = 3; ~1 μg) were separated using SDS-PAGE on gradient (4–15%) Mini-PROTEAN1TGX™ Precast Gels (Bio-Rad, Hercules, CA, United States) and transferred to the membrane using a Trans-Blot Turbo Transfer System (Bio-Rad, United States). Membranes were blocked in 5% nonfat milk in 1× Tris-buffered saline-Tween 20 (TBS-T) buffer for 2 h at room temperature. The membranes were probed with ALIX, ompA, and LPS antibodies (Sigma, St. Louis, MO, United States) overnight at 4°C. The membrane was incubated with an appropriate peroxidase-conjugated IgG secondary antibody for 1 h at room temperature. According to the manufacturer’s recommendations, all blots were developed using ECL chemiluminescence reagents and a western Blotting Detection System (Amersham, Piscataway, NJ, United States).

#### 2.3.5. ExoView analysis

Exosomal tetraspanin marker proteins were detected with an ExoView analysis described previously (Radnaa et al., 2021a). Briefly, 35 μL of EVs (5 × 10^6^/chip) were diluted in solution A (NanoView Biosciences) and incubated on tetraspanin microarray chips in a 24-well plate overnight at RT. Each chip was pre-coated with CD9, CD63, CD81 antibodies, and MIgG control antibodies. The next day unbound EVs were washed three times for 3 min at a 500 rpm shaker in solution A. Then, EVs were co-stained with CD81, CD9, and CD63 antibodies diluted in blocking solution (NanoView Biosciences) for 1 h at RT in the dark. The tetraspanin microarray chips were then sequentially washed three times for 5 min in solution A, in solution B (NanoView Biosciences), and three times for 5 min in Milli-Q water (ELGA) in a 500 rpm shaker. Then, the chips were imaged on the ExoView R100 instrument (NanoView Biosciences) using the ExoView Scanner 3.1.4 acquisition software. The size distribution and concentration were determined using ExoView Analyzer 3.1.4 (NanoView Bioscience).

### 2.4. Microbiome characterization

#### 2.4.1. Placental DNA extraction

Placental tissue pieces were collected as mentioned above and stored at −80°C freezer until genomic DNA extractions. Tissue DNAs were extracted according to the manufacturer’s instructions in a disinfected laminar flow hood (Qiagen, Hilden, Germany, Cat. No: 69506). Briefly, small pieces of tissues (approximately ≤25 mg) were placed in sterile Eppendorf tubes (free of DNAse/RNAse), and 180 μL of Lysis Buffer (Buffer ATL) was added. Then 20 μL of proteinase K was added to the tube, vortexed, and incubated at 56°C until tissues were lysed. 200 μL of AL Buffer was added, vortexed, and incubated at 56°C for 10 min. DNA was then extracted per the kit instructions with a final elution in 100 μL of Buffer AE. Isolated DNA concentrations were measured by Spectrophotometry (BioTek Synergy H4 Hybrid Reader) and stored at −80°C freezer.

#### 2.4.2. BEV DNA isolation

Bacterial extracellular vesicle-derived DNAs were isolated using a GenElute™ Bacterial Genomic DNA Kit (Sigma-Aldrich, Cat. No: NA2100) according to the manufacturer’s instructions. Briefly, 180 μL of Lysis Solution T was added to 100 μL isolated BEVs (1.5E+11 ± 2.4E+10) and incubated at RT for 2 min. Then, 20 μL of Proteinase K was added, vortexed, and incubated at 55°C for 30 min. 200 μL of solution C was added, vortexed and incubated at 55°C for 10 min. The binding column was prepared by adding 500 μL of kit-provided column preparation solution and centrifuged at 14,000 rpm. Then 200 μL of ethanol was added to the lysed BEVs, vortexed, and directly added to the prepared binding column. After centrifugation at 8,000 rpm for 1 min, the column was washed with 500 μL of wash solution twice. Finally, BEV DNA was eluted in 200 μL of elution solution. Isolated BEV DNA concentrations were determined by spectrophotometry (BioTek Synergy H4 Hybrid Reader) and stored at −80°C freezer.

#### 2.4.3. 16S sequencing

High throughput sequencing of the bacterial 16S rRNA gene was performed using DNA extracted from the BEV and placenta samples. Sequencing libraries for each isolate were generated using the Quick-16S Plus NGS Library Prep Kit (D6420, Zymo Research, Irvine, CA, United States), which targets the 16 s rRNA V3-V4 hypervariable regions using the priamre pair 341f (CCTACGGGDGGCWGCAG, CCTAYGGGGYGCWGCAG, 17 bp) and 806r (GACTACNVGGGTMTCTAATCC, 24 bp). Modification to the manufacturer protocol included the use of 3 μL of extracted DNA as the starting material for library preparation. A single qRT-PCR was performed for 42 cycles to amplify the target V3-V4 region and incorporate barcoded adapters compatible with the Illumina flow cell using the LightCycler 480 Instrument II (Roche Sequencing and Life Sciences, Indianapolis, Indiana, United States). DNA extracted from *E*. *coli*, and isolation reagent buffer mix served as positive and negative controls, respectively, for the DNA extraction process. ZymoBIOMICS Microbial Community DNA Standard and ZymoBIOMICS DNase/RNase Free water served as positive and negative controls, respectively, for the sequencing library preparation protocol. Equal volumes of the qRT-PCR products were pooled together (5 μL each) with 50 μL of PCR inactivation solution (D6420 kit reagent, Zymo Research, Irvine, CA, United States). A single library clean-up was done on the pooled samples using the Select-a-Size Magbead solution (D6420 kit reagent, Zymo Research, Irvine, CA, United States) with a final elution volume of 20 μL. Size verification of pooled library and a random set of individual libraries was performed using the Agilent Bioanalyzer DNA 1000 kit (5067–1504, Agilent Technologies, Santa Clara, CA, United States). Pooled library concentration was measured on a Qubit 3.0 fluorometer (Q33216, Invitrogen, Waltham, MA, United States) using 2 μL of pooled library incubated for 2 min with 198 μL of Qubit 1x dsDNA High Sensitivity Assay working solution (Q33231, Invitrogen, Waltham, MA, United States).

The library was then diluted to 4 nM using 10 mM Tris–HCl pH 8.5 (T1062, Teknova, Hollister, CA, United States). Denatured library for sequencing was prepared by incubating 5 μL of 4 nM pooled library with 5 μL of 0.2 N NaOH (SS255-1, Fisher Scientific, Waltham, MA, United States) for 5 min at room temperature, then adding 990 μL of pre-chilled HT1 hybridization buffer (20015892, Illumina, San Diego, CA, United States) to make 20 pM library solution. Concomitantly, a 10 nM PhiX calibration and diversity control (FC-110-3001, Illumina, San Diego, CA, United States) was diluted to 4 nM using 10 mM Tris–HCl, pH 8.5 with 0.1% Tween 20 (T7724, Teknova, Hollister, CA, United States). The resulting 4 nM PhiX was denatured and diluted to 20 pM using the same protocol applied to the pooled library. The denatured library and PhiX solutions were further diluted to 12 pM each by adding 240 μL of pre-chilled HT1 buffer to 360 μL of 20 pM pooled library or PhiX control. To ensure efficient denaturation of the sequencing templates before loading to the MiSeq reagent cartridge, a final library solution composed of 120 μL of 12 pM PhiX control and 480 μL of 12 pM pooled library was denatured in a heat block set at 96°C for 2 min, inverted twice to mix, and immediately immersed in an ice bath for 5 min. Sequencing was performed on an Illumina MiSeq instrument using a MiSeq 600-cycle paired-end reagent kit v3 (MS-102-3003, Illumina, San Diego, CA, United States).

High-throughput data generation platforms, like second-generation sequencing, are susceptible to batch effects due to run-to-run variation in reagents, equipment, protocols, and personnel. All the samples in each group were processed using the same nucleic acid isolation kits and validated library preparation kits. All the BEV and nucleic acid isolations reagents were included as a negative control to account for potential contamination. Mock microbial community and negative controls were included as part of the sequencing library preparation. Any OTUs which appeared in the negative controls were excluded from any analysis. All the samples underwent library preparation in the same batch and were sequenced on a single sequencing run.

#### 2.4.4. Metagenomic DNA characterization

To identify known bacteria, sequences were analyzed using the CLC Genomics Workbench 23.0.3 Microbial Genomics Module (CLC MGM). Reads containing nucleotides below the quality threshold of 0.05 (using the modified Richard Mott algorithm) and those with two or more unknown nucleotides or sequencing adapters were trimmed out. Reference-based Operational Taxonomic Unit (OUT) picking was performed using the SILVA SSU v138.1 99% database (Quast et al., 2013). Sequences present in more than one copy but not clustered to the database were placed into *de novo* OTUs (99% similarity) and aligned against the reference database with an 80% similarity threshold to assign the “closest” taxonomical name where possible. Chimeras were removed from the dataset if the absolute crossover cost was three using a k-mer size of 6. OTUs with a combined abundance of less than 2 were removed from the analysis. Low abundance OTUs were removed from the analysis if their combined abundance was below 10 or 0.01% of reads. Abundance profiling was performed using MicrobiomeAnalyst (Chong et al., 2020) at various taxonomic levels using actual and total sum scale abundance. Post-initial abundance profiling, the OTUs found in the reagent control sample were discarded from all other samples to exclude the ambiguity of whether this was found due to contamination. All additional analysis was performed post-subtraction of the host OTUs with total sum scaling.

Alpha diversity was measured using the observed number of features and Shannon entropy at the genus level to describe the identified metagenomic richness and evenness. Beta diversity was calculated using the Bray–Curtis dissimilarity measure (genus level). Permutational Diversity analysis was performed using MicrobiomeAnalyst and visualized with GraphPad Prism 9. Multivariate ANOVA (PERMANOVA) analysis was used to measure effect size and significance on beta diversity for grouping variables (Anderson and Santana-Garcon, 2015). Heatmaps were generated using the median ratio (log2) with hierarchical clustering on the genera by their Euclidian distance using Ward’s method (Murtagh and Legendre, 2014; Supplementary Figure S2). Univariate statical comparisons were performed between the BEV and placental samples at the genus level using Mann–Whitney/Kruskal-Wallis methods with an FDR value of *p* cut-off of 0.05. Additionally, differential abundance analysis was performed using CLC MGM at the genus level to compare the differences between the two samples classes while correcting for the patient. The tool models each OTU as a separate generalized linear model, where it is assumed that abundances follow a negative binomial distribution. The Wald test was used to determine significance between groups. The CLC MGM analysis used trimmed mean of *M*-values instead of total sum scaling and a Bonferroni corrected value of *p* cutoff of 0.05.

### 2.5. Functional effects of placental BEV in human maternal and fetal cells

#### 2.5.1. Placental BEV uptake in human BeWo and decidual cells

Isolated BEVs were labeled with DiOC18(3) (3,3′-dioctadecyloxacarbocyanine perchlorate—DiO; D275; Invitrogen, Carlsbad, CA) as previously described with minor modifications (Radnaa et al., 2021a). Briefly, 100 μL of BEVs were incubated with 10 μM DiO at 37°C for 30 min. After the incubation, 50 μL of 5% BSA was added to the DiO-labeled BEVs. The BEV suspension was transferred to an Amicon Ultra-15 Centrifugal Filter Unit (UFC910024, 100 kDa; Merk Millipore Ltd., Tullagreen, Carrigtwohill, Co. Cork, Ireland) in addition to 5 mL of PBS and was centrifuged at 4,000 × g for 10 min to remove excess DiO. Approximately, 30,000 BeWo and 20,000 hFM-DEC cells were seeded on eight-well coverslips (CELLTREAT Scientific Products, Pepperell, MA, United States, Cat. No: 229168) for BEV cellular uptake by immunocytochemistry (total *N* = 4 for each condition). A total of 9 × 10^8^ DiO-labeled BEVs were added to each well, and cells were incubated at 37°C, 5% CO_2,_ and for 24 h.

#### 2.5.2. Immunocytochemical staining

After 24 h of DiO-labeled placental BEV incubation, the medium was removed, and cells were fixed in 4% paraformaldehyde. After fixing, non-specific binding was blocked by incubation in 3% BSA/PBS for 30 min at RT. Cells were then incubated overnight at 4°C with primary antibodies for cytokeratin-7 (Abcam, Cat. No: ab9021, DF: 1:300 dilution) or alpha-smooth muscle actin (Invitrogen, Cat. No: 14-9760-82, DF: 1:300). The next day, the cells were washed three times for 10 min with 1 × PBS and incubated with appropriate Alexa Fluor secondary antibodies (Alexa Fluor® 594, Invitrogen, Cat. No: ab150080) diluted in PBS for 1 h at RT. After that, the cells were stained with DAPI (Invitrogen), washed three times for 10 min, and then mounted using Mowiol mounting medium (475904–100GM-M; Sigma-Aldrich).

#### 2.5.3. *Escherichia coli* culture and BEV isolation

The pathogenic strain of bacteria used in this study was ATCC 12014 *Escherichia coli* O55:K59(B5):H obtained from Remel Laboratory of Thermo Fisher (Thermo Fisher Scientific, Remel Products, Lenexa, KS, United States, Lot # 496291). The bacteria were cultured in sterile, non-selective nutrient broth (BD Biosciences) and stocks were stored at −80°C in 20% glycerol. The logarithmic phase *E*. *coli* culture was used to isolated BEV using ExoBacteria™ OMV Isolation Kit for *E*. *coli* and other gram-negative bacteria (Cat# EXOBAC100A-1, System Biosciences, Palo Alto, CA, United States).

#### 2.5.4. Human THP-1 macrophages, BeWo, and decidual cell cultures

THP-1 monocytes were obtained from the American Type Culture Collection (ATCC TIB-202, Manassas, VA, United States) and were cultured in RPMI 1640 Medium (ATCC modification; A1049101, Thermo Fisher Scientific, Waltham, MA, United States) and supplemented with 0.05 mM 2-mercaptoethanol (21985023, Gibco™, Carlsbad, CA, United States) and 10% heat-inactivated FBS (Sigma-Aldrich, F2442, St. Louis, MO, United States). The cells were grown until they reached a density of 1 × 10^6^ cells/mL. The cells were then centrifuged at 1,000 rpm for 5 min. THP-1 monocytes were seeded in a 48-well plate (50,000 cells per well). They were differentiated to THP-1 macrophages by incubating in a medium containing 100 mM phorbol 12-myristate 13-acetate for 3 days at 37°C and 5% CO2 and grown to 80% confluence as described previously (Tantengco et al., 2021a).

BeWo cells (ATCC CCL-98) were used to represent placental cytotrophoblasts and were cultured in DMEM/F12 (Gibco, #10–092-CV) medium plus 15% heat-inactivated FBS (Sigma), 1% penicillin/streptomycin, 1% amphotericin B (Sigma, A2942) at 37°C, and 5% CO_2_ until 80% confluence was reached. Human fetal membrane-derived maternal decidua parietalis cells (hFM-DECs; Radnaa et al., 2021b) were grown in complete medium containing DMEM/F12 (Gibco, #10–092-CV), 10% heat-inactivated FBS (Sigma, F2442), 1% penicillin/streptomycin, 1% amphotericin B (Sigma, A2942) at 37°C, 5% CO_2_ until 80% confluence was reached. For experimental studies, each media formulation was created with EVs removed FBS (Hadley et al., 2018). All cultures were confirmed to be free of detectable *Mycoplasma* spp. contamination by a commercial PCR kit (MycoSensor qPCR Assay Kits, Santa Clara, CA, United States, Cat. No: 302106).

#### 2.5.5. Placental BEV treatment of human THP-1 macrophages, BeWo, and decidual cell

Extracellular vesicles-free culture media were used for all placental BEV treatments. To treat the cells with human placenta-derived BEVs, THP-1 monocytes were seeded in a 48-well plate (50,000 cells per well). They were differentiated to THP-1 macrophages by incubating in a culture medium containing 100 mM phorbol 12-myristate 13-acetate for 3 days at 37°C and 5% CO2 and grown to 80% confluence as described previously (Tantengco et al., 2021a). The next day, the medium was removed, washed with 1× PBS, and replaced with the EVs-free medium. Approximately, 9 × 10^6^ BEVs per well (low dose, *n* = 5 wells) and 9 × 10^8^ BEVs per well (high dose, *n* = 5 wells) were added. The cells were incubated with the BEVs for 24 h. Similarly, BeWo (60,000 cells) and hFM-DEC (40,000 cells) were seeded in 24-well plates and grown overnight. After 24 h, the media was replaced with EVs-free equivalents, and 9 × 10^6^ BEVs were added to each well (*n* = 5). For each cell type, five wells were treated with the EVs-free medium as a negative control. In parallel, five wells were exposed to *E*. *coli*-derived BEVs at a concentration of 9 × 10^6^ BEVs/well as a positive control. The cells were incubated for 24 h; media was collected from each well and stored at −80°C for enzyme-linked immunosorbent assay.

#### 2.5.6. Enzyme-linked immunosorbent assay

The collected culture media from all treatments and cell types were analyzed using an enzyme-linked immunosorbent assay for IL-1 (BD OptEIA™ Human IL-1 ELISA Set—557953, BD Biosciences, IL-6; BD OptEIA™ Human IL-6 ELISA Set—555220, BD Biosciences, DF: 1:2), and IL-10 (BD OptEIA™ Human IL-10 ELISA Set—555157, BD Biosciences), as per the kit instructions. For each ELISA, duplicate standards were used to create a dilution series according to the manufacturer’s recommendations. The assay results were analyzed using a Synergy H4 microplate reader (BIO-TEK).

### 2.6. Statistical analyses

The cytokine data were analyzed using Prism 9 software (GraphPad Software, La Jolla, CA, United States). The Shapiro–Wilk test for normality was conducted to assess the data. The Student’s *t*-test was used to compare results with two means. Ordinary one-way ANOVA followed by Tukey’s multiple comparison test was used to compare normally distributed data with at least three means. The Kruskal–Wallis test with Dunn’s multiple comparison test was used for data that were not normally distributed. Asterisks denote *p* values as follows: *p < 0.05, **p < 0.01, ***p < 0.001, and ***p < 0.0001.

## 3. Results

### 3.1. EVs and BEV quantification and characterization

**Figure 2:**
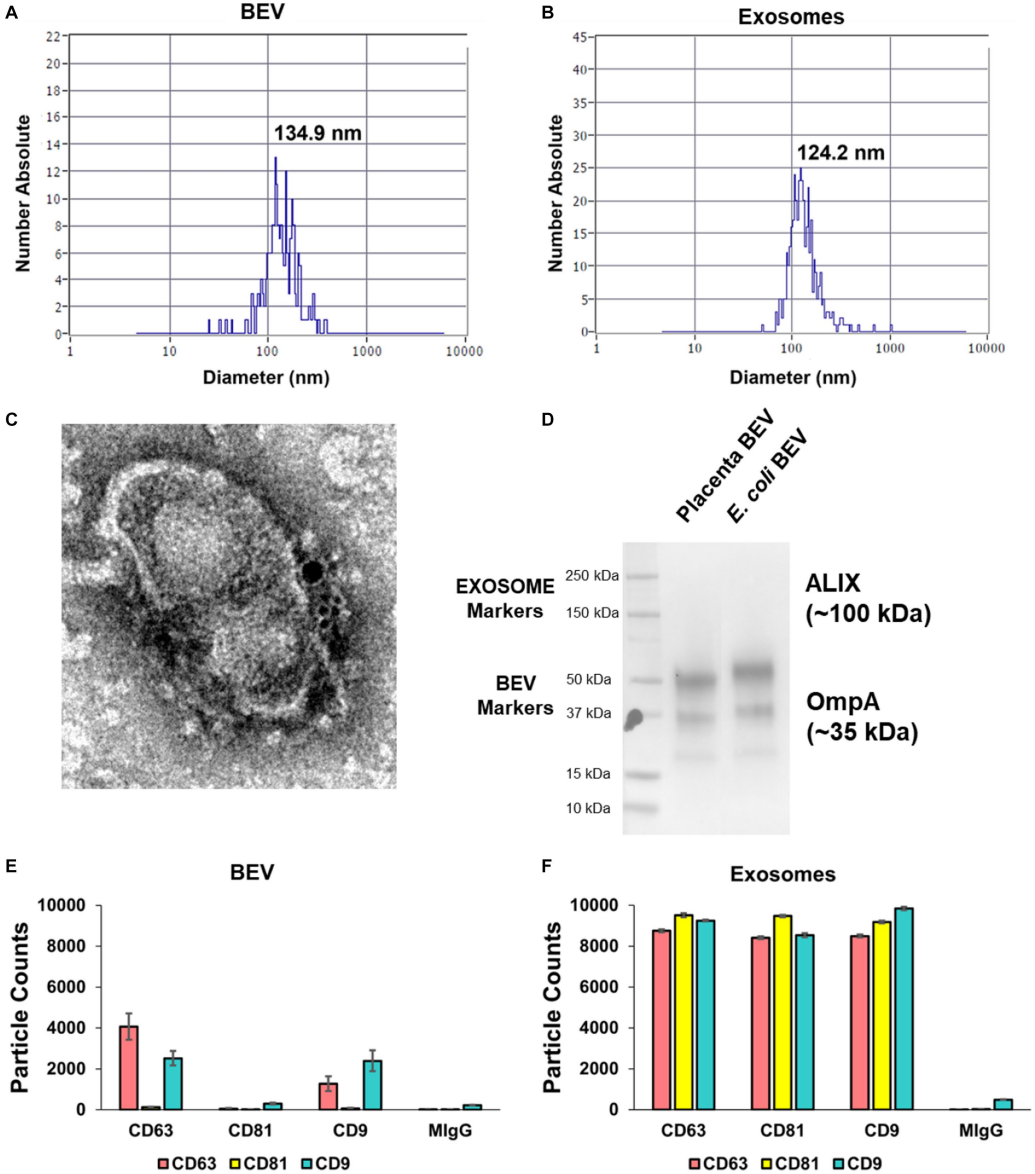
Characterization of isolated BEV from human placental tissues and control human extracellular vesicles (EVs). Nanoparticle tracking analysis demonstrating the size distribution of BEV **(A)** and control CTC-derived EVs **(B)**. The average size of BEVs was 134.9 nm, and the mean concentration was 1.8–5.5 × 10^11^ BEVs/g of the wet placenta. **(C)** Immunogold transmission electron microscopy of BEV. The yellow arrow shows the OmpA (15 nm gold) while the red arrow shows the LPS (6 nm gold) at the surface of the BEV. Scale bar 100 nm. **(D)** Co-immunoprecipitation and pull-down assay showing the OmpA protein was expressed in BEV from the placenta and from *E*. *coli* (positive control). The isolated BEV samples from the placenta and *E*. *coli* were negative for EVs marker, ALIX. **(E,F)** ExoView analysis on human placenta derived BEV. EVs tetraspanin marker proteins: CD9, CD63, and CD81 expressions were displayed in ExoView counts. MIgG spot was used for negative control. The data are presented as the means ± SEM. Three technical replicates were used. BEV, bacterial extracellular vesicles. CTC, chorion trophoblast cells.

Using published protocols and experiences with human and bacterial samples in our lab, we empirically optimized a method that extracted isolated BEVs and EVs from placental tissues. Using the optimized method, we processed 35 placentas from scheduled, uncomplicated C-sections to provide fresh material for subsequent characterization studies. To confirm the sterility of the BEV samples, 20% of each sample was plated onto LB-agar and observed over 72 h of incubation. No bacterial growth was observed in LB agar from any BEV samples (Supplementary Figure S3). BEVs were characterized for their size distribution after the isolation (Figure 1) with a nanoparticle tracking analysis (NTA; Figure 2A). Chorion trophoblast cells (CTC)-derived EVs were used as positive controls (Figure 2B). The median size of the BEVs was 134.9 nm (Figure 2A), and control EVs were 124.2 nm (Figure 2B). The BEV concentration in wet placenta was 1.8–5.5 × 10^11^ BEVs/g. Negative staining and immunogold transmission electron microscopy showed the presence of ompA (15 nm gold) and LPS (6 nm gold) at the surface of the BEV (Figure 2C; Supplementary Figure S4). Co-immunoprecipitation and pull-down assay showed that OmpA protein was expressed in BEV from the placenta. *Escherichia coli*, the positive control, also expressed OmpA. The isolated BEV samples from the placenta and *E*. *coli* were negative for EVs marker ALIX (Figure 2D). Next, we analyzed the exosomal tetraspanin marker protein expressions on the BEV surfaces by comparing them with control EVs (Figures 2E,F). As expected, CD81 expression was not detected in BEV samples (Figure 2E), and CD63 and CD81 were detected scarcely compared to control EVs (Figure 2F).

### 3.2. Metagenomic 16S analysis

We next assessed the profile of the 16S rRNA gene sequences in placental and BEV DNA samples using V3-V4 sequencing using 341f and 806r primers because they are expected to detect 84.2% of bacteria and 75.8% of archaea (Klindworth et al., 2013). The V3-V4 region is relatively long (typically ~ 460 bp), increasing the confidence in the specificity of microbe detection as previously reported (Urushiyama et al., 2017; Parnell et al., 2017a; de Goffau et al., 2019; Dinsdale et al., 2020; O'Callaghan et al., 2020; Theis et al., 2020a; Sterpu et al., 2021; Turunen et al., 2021). Using standardized methods, we amplified and sequenced the sample set, and additional controls, including reagent buffers used for sample preparation (2), DNase/RNase-free water, *E*. *coli* DNA, and ZymoBIOMICS Microbial Community DNA Standard (1) encoding divergent microbial 16S rRNA gene sequences that all (2) underwent the same isolation procedures. This approach verified the robust performance of biochemical steps and proper sample tracking. We recovered useable data (≥1,000 reads) for 59 of 59 placental or BEV samples and the six controls for a total of 10,041,326 paired reads completed in a single Illumina flow cell run. The output 16S rRNA gene sequences were clustered using CLC MGM, and clusters were assigned taxonomically using the SILVA v138.1 99% database.

**Figure 3:**
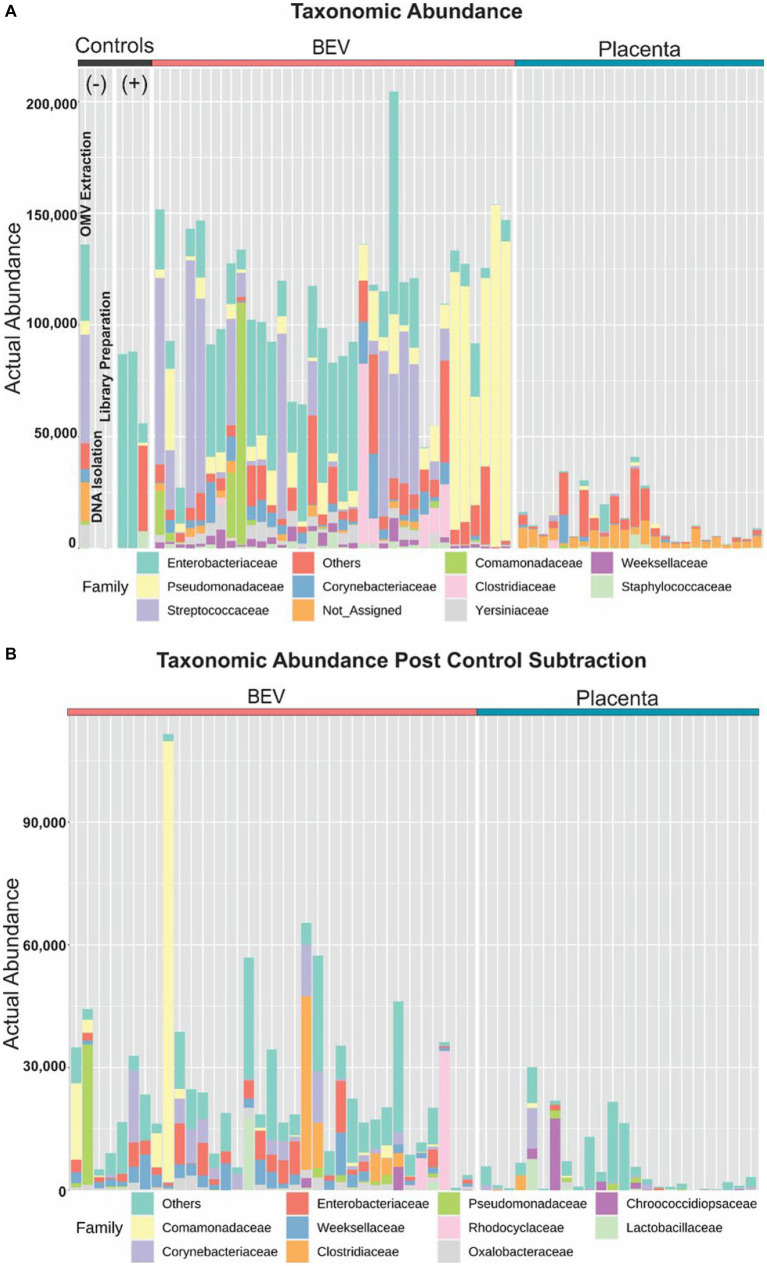
Profiling taxonomic abundance. **(A)** Sequencing of the placental samples yielded very few, or no reads classified as bacterial 16S rRNA database, whereas microbial DNA was identified in BEVs derived from the same placental samples. We observed microbial signatures in the pool of reagents used for BEV isolation and assays. BEVs isolated from *Escherichia coli* were used as a positive control. **(B)** After removing the contaminants or operational taxonomic unit reduction, BEV samples have microbial signatures beyond contaminants whereas the placenta had minimal microbial signatures.

**Figure 4:**
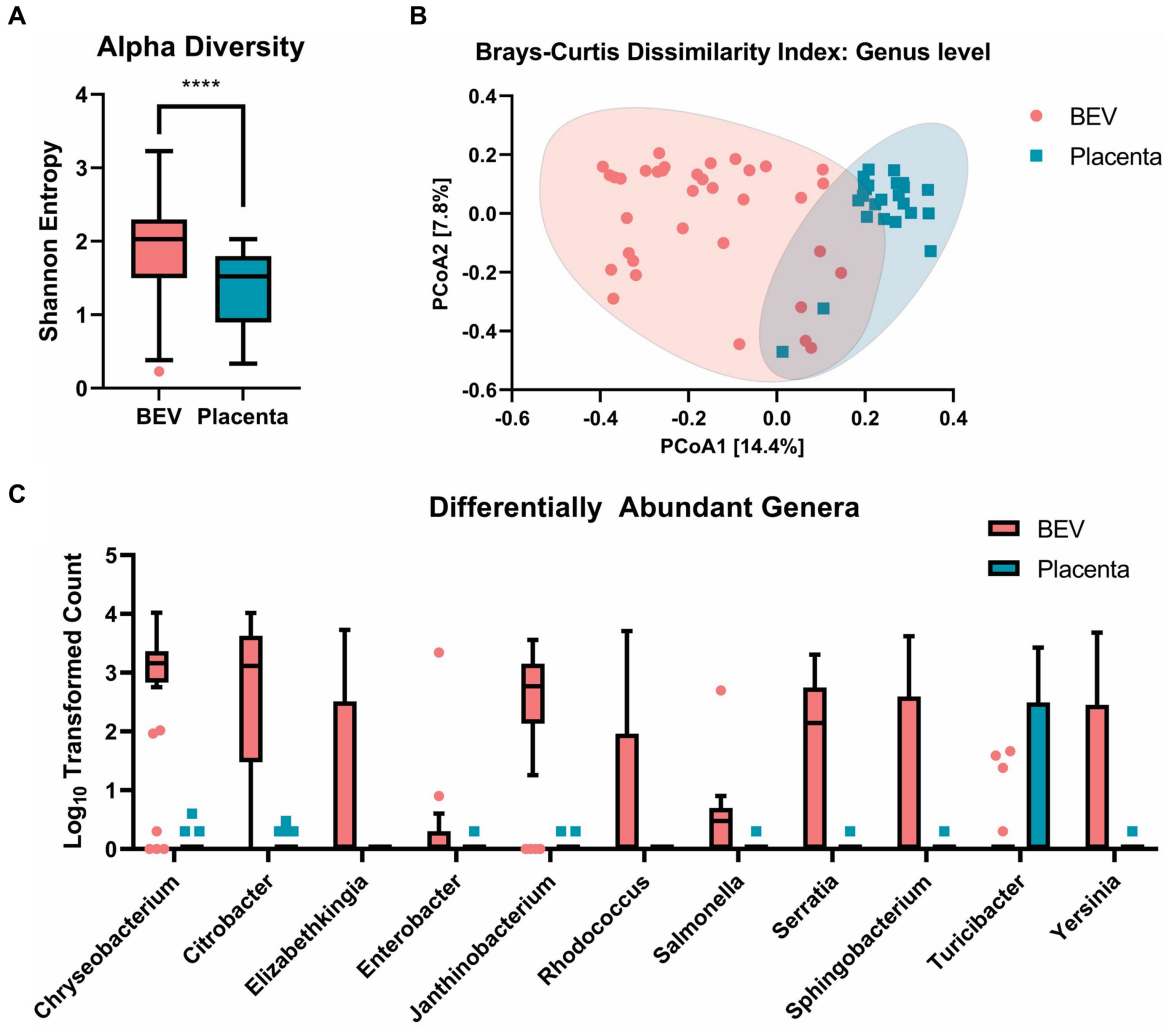
**(A)** a-diversity: Shannon entropy comparison at genus level between BEV and placenta samples, showing that BEV samples are significantly more even and rich (*p* < 0.0001). **(B)** b-diversity: beta diversity shows a significant overlap between placental and BEV DNA, indicating a similar microbiome community between the BEV and placenta samples. **(C)** Log_10_ transformed count data of genera identified as differentially abundant in the placental tissues and BEV using univariate analysis. The genera shown were identified as statistically significant using Mann–Whitney/Kruskal-Wallis non-parametric statistical tests with a cut-off of FDR value of *p* ≤ 0.05.

The major lineages detected are summarized in Figure 3 and detailed further in the supplemental Operational Taxonomic Unit Table. BEV and Placental samples showed well-known bacterial lineages associated with different organ systems in the human body. The negative controls contained environmental bacteria commonly associated with contaminants, such as *Streptococcus* and *Enterobacter*. A total of 4,111 OTUs were predicted in the sequenced samples, with 3,467 found in the SILVA database and 644 “*de novo*.” However, upon further inspection by *blastn* against the nucleotide collection database, the “*de novo*” OTUs were classified as amplified host materials and were excluded from further analyses. Any operational taxonomic units (OTU) found in either the reagent control or the sequencing control samples were removed from all downstream analyses, as summarized in Figure 4. The average number of reads assigned to OTUs was 30,509 and 7,945 for BEV and placental samples (after filtration and subtraction of controls), respectively. After filtration criteria, 34 and 17 samples contained ≥1,000 reads assigned to OTUs for BEV and placental samples, respectively.

We observed significantly higher richness and evenness of the bacterial lineages in the BEV samples compared to the placental samples by Shannon-Entropy index (Figure 4A). Clustering of the samples using Brays-Curtis showed a lack of clear separation of the placental and BEV samples, indicating that most of the bacterial lineages found in the placental samples were also present in the BEV samples (Figure 4B). To investigate this further, we performed differential abundance analysis between the two groups and found that 10 genera (i.e., *Chryseobacterium*, *Elizabethkingia*, *Enterobacter*, *Citrobacter*, *Janthinobacterium*, *Yersinia*, *Salmonella*, *Serratia*, *Sphingobacterium*, and *Rhodococcus*) were overrepresented in the BEV samples compared to placenta samples, and one genus was overrepresented in the placenta samples compared to the BEV samples (Figure 4C). *Turicibacter* was identified in 10 placental samples but was only detected in four BEV samples. *Turicibacter* is a genus in the Bacillota phylum of bacteria and is usually present in animals’ gastrointestinal tracts. Additionally, the CLC MGM differential abundance analysis utilizing a subset of the patient-matched BEV, and placenta samples showed 39 genera (with a max group mean ≥ 100 reads and Bonferroni value of *p* ≤ 0.05) to be differentially between the two groups when controlling for the patients. *Turicibacter* was not identified as differentially detected in the secondary analysis. The results of both differential abundance analyses are presented in Supplementary Table S1 and the species-level haplotype in Supplementary Table S2. These results showed the microbial sources of BEVs isolated from human placenta.

### 3.3. BEV uptake in human decidual and placental cells

**Figure 5:**
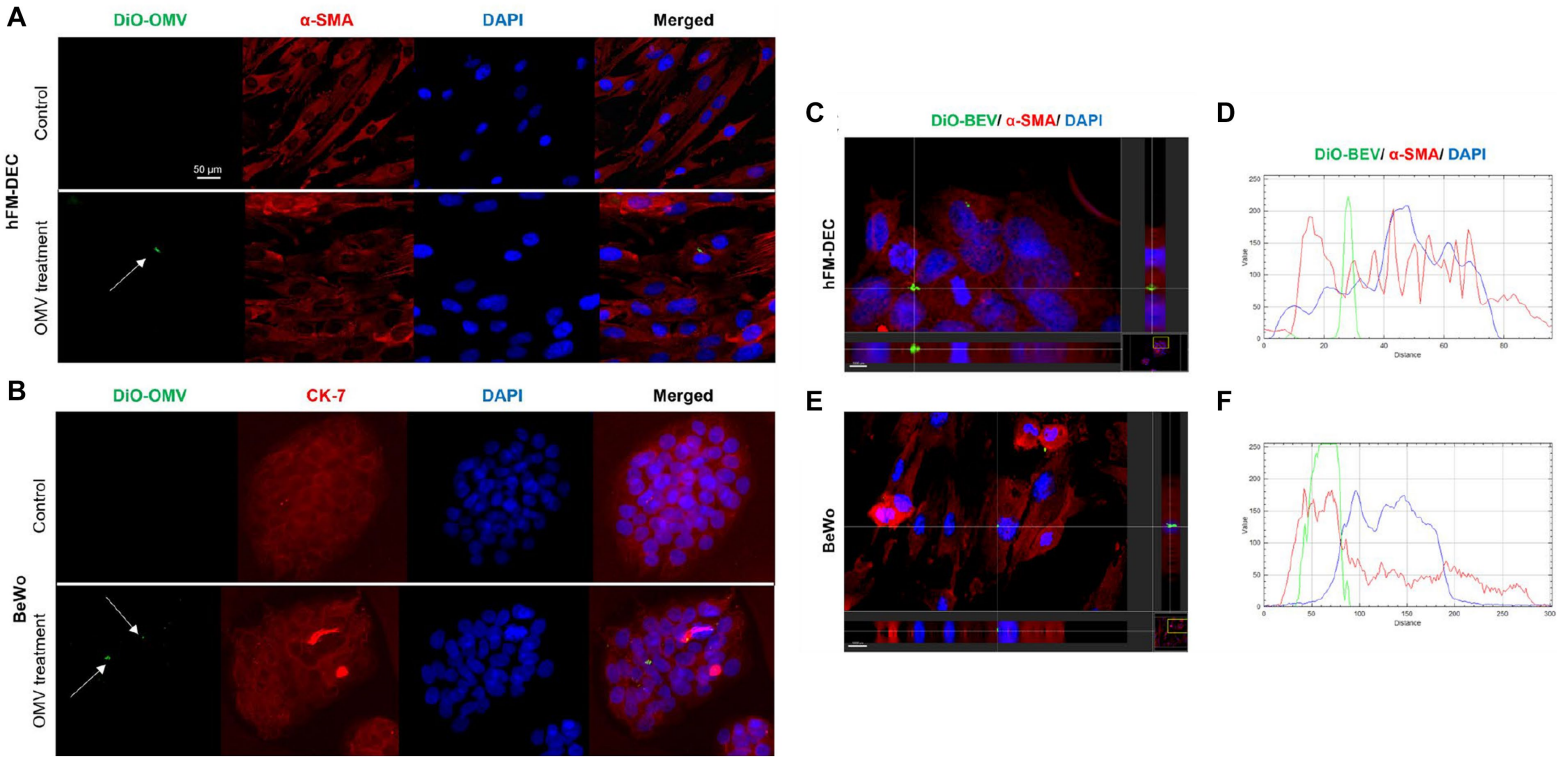
Human placenta derived BEVs cellular uptake. Fluorescence microscopy imaging showing DiO-labeled BEV (green-white arrow) in hFM-DEC (α-smooth muscle actin-red; **A**) and BeWo (CK-7-red; **B**) after 24 h treatment (9 × 10^8^ BEVs per well). Nuclei are stained with DAPI. Scale bar, 50 μm. Magnification, 20×. hFM-DEC (scale bar 5,000 μm, magnification 40×) and BeWo (scale bar 10,000 μm, magnification 20×) section from IMARIS software. IMARIS slice of fluorescence microscopy imaging showing DiO-labeled BEV (green-white arrow) and the topographical profile of the pixel intensity levels of showing the colocalization of DiO-labeled BEV (green line) inside hFM-DEC **(C,D)** and BeWo (**E,F**; red line) after 24 h of treatment, scale bar 2,000 μm.

The uptake of placental-derived BEVs was confirmed using fluorescence microscopy and z-stack analysis. Dio-labeled BEV and BeWo/ hFM-DEC cell-specific markers were used. As shown in Figures 5A,B, there was an uptake by both types of cells of DiO labeled BEVs (green dots) after 24 h of treatment. As expected, the control group did not have DiO markers in the cells. Slice and section images from IMARIS software (Figures 5C–F) showed that DiO-labeled placental-derived BEVs are located inside the cells. There was no significant difference in the amount of placental-derived BEVs taken up by beWo and hFM-DEC cells (Supplementary Figure S5). These results showed that placental-derived BEVs can be taken up by maternal and fetal cells.

### 3.4. Determination of BEV-mediated cytokine response in THP-1 macrophages, decidual and placental cells

**Figure 6:**
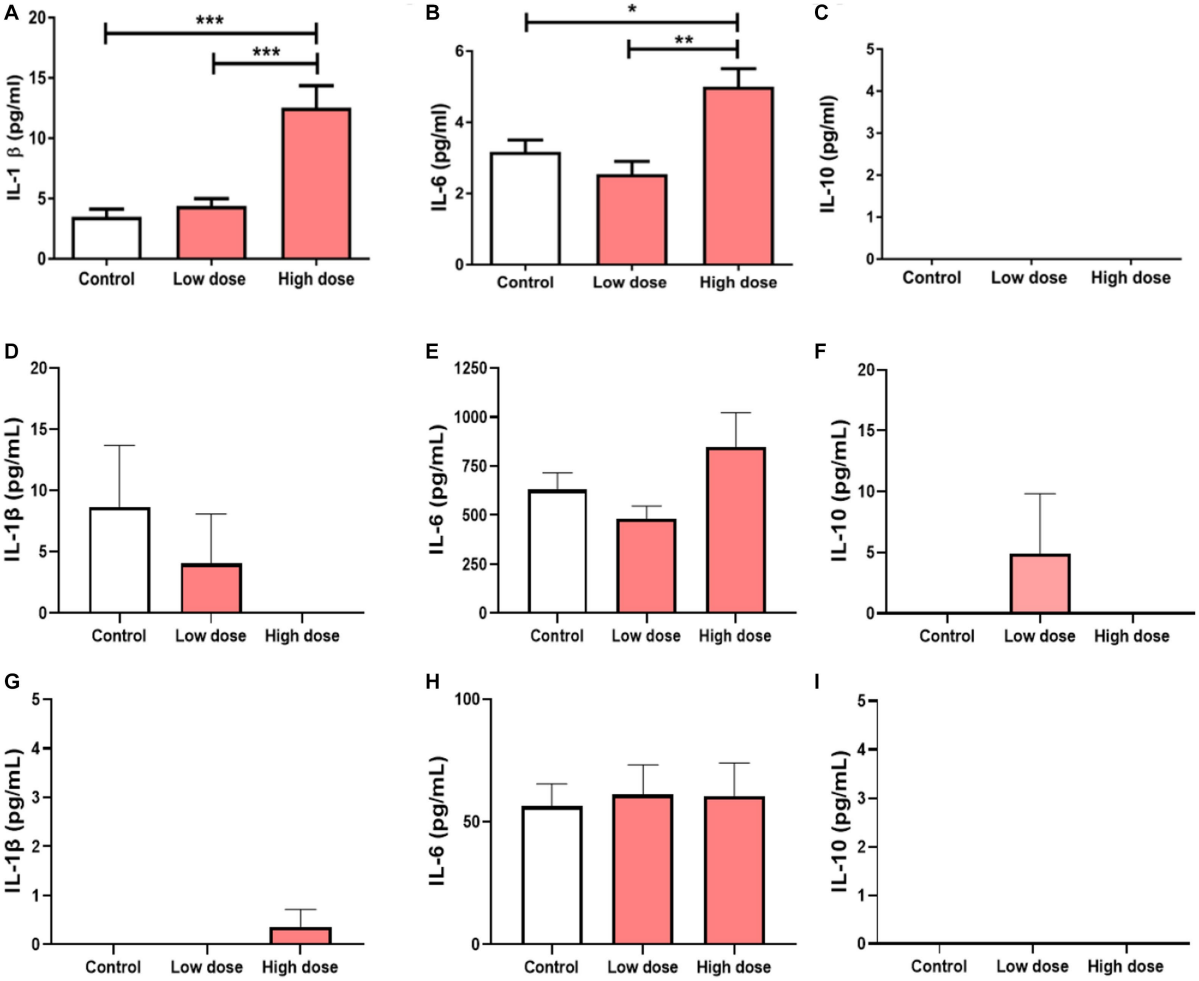
Cytokine concentrations in THP-1 macrophages after 24 h incubation with BEV from human placenta (low dose: 9 × 10^6^ BEVs; high dose: 9 × 10^8^ BEVs). The concentrations of IL-1β, IL-6, and IL-10 were measured. The values represent mean ± SD (*n* = 5). *p < 0.05 **(A–C)**. Cytokine concentrations in maternal hFM-DEC cells **(D–F)** and BeWo cells **(G–I)** after 24 h incubation with BEV from human placenta (low dose: 9 × 10^6^ BEVs; high dose: 9 × 10^8^ BEVs). The concentrations of IL-1β, IL-6, and IL-10 were measured. The values represent mean ± SD (*n* = 5). *p < 0.05; **p < 0.01; ***p < 0.001.

To test the functional effects of BEVs isolated in the placenta in immune cells, THP-1 monocytes differentiated into macrophages were treated with low- or high-dose BEVs for 24 h. ELISA results showed that only high-dose placental BEVs treatment significantly increased IL-1β and IL-6 (all *p* < 0.05) in THP-1 macrophages (Figures 6A–C) compared to medium alone controls. However, placental BEV treatments did not increase the inflammatory cytokines released by decidual (Figures 6D–F) and BeWo cells (Figures 6G–I).

To test if BEVs from pathogenic bacteria can promote inflammatory response, we also performed cytokines analysis using *E*. *coli* derived BEVs as a positive control in maternal decidua and placental cells (Supplementary Figure S5). IL-6 levels were significantly increased in hFM-DEC cells after treatment with low-dose of BEVs from *E*. *coli* compared to controls (*p* < 0.01), whereas BeWo cells showed no significant change in inflammatory cytokines (Supplementary Figure S6). Interestingly, high-dose of *E*. *coli*-derived BEVs were toxic to both cell types and cells were completely dead after 24 h of treatments. Therefore, inflammatory cytokines were not measured for high-dose *E*. *coli*-BEVs treatments.

We also compared the functional effects of placental-derived BEVs and *E*. *coli*-derived BEVs. The data showed that decidua cells had significantly higher IL-6 levels (*p* < 0.01) and BeWo cells had higher IL-1β levels (*p* < 0.05) when treated with *E*. *coli*-derived BEVs than with placental BEVs. On the other hand, BeWo cells had lower IL-6 levels (*p* < 0.05) when treated with *E*. *coli*-derived BEVs than with placental BEVs (Supplementary Figure S7). These results indicate that placental-derived BEVs did not induce inflammation in maternal and fetal cells. The specific functions of these BEVs are still unknown and require further investigation. However, it was observed that BEVs derived from the pathogenic bacterium *E*. *coli* triggered an inflammatory response in both maternal and fetal cells.

## 4. Discussion

Our study successfully isolated bacterial extracellular vesicles (BEVs) from human tissue (placenta). This study used orthogonal biophysical separations, which involved ultrafiltration, size-exclusion chromatography, and density-gradient centrifugation. This technique was used in isolating BEVs from human body fluids, such as blood and stool (Tulkens et al., 2020). This was modified and adapted to placental tissues. There is a commercially available kit for isolating BEVs that utilize innovative precipitation-free, ion-exchange chromatography system. However, this system was only optimized for isolating BEVs from bacterial culture medium. Hence, this was only used in isolating BEVs from *E*. *coli* cultures in this study.

The placenta contains BEVs with microbial DNA beyond the threshold of a stochastic noise based on the reagent controls included in our studies. Placental BEVs exhibited reported characteristics (size and shape,) and the markers expressed indicated the predominance of Gram-negative BEVs over Gram-positive bacterial extracellular vesicles. The quantity of BEVs isolated was similar to the number of EVs (extracellular vesicles of 30–160 nm) normally quantitated in maternal plasma. Our BEV preparations had minimal detection of tetraspanin markers expected on human cell derived EVs confirming both negligible human material and EV contamination and the bacterial origin of the BEVs. Compared to DNA isolated directly from placental tissue, BEVs had a significantly higher number of detected genera with greater relative abundance after total sum scaling. Interestingly only a single genus showed discordant presence in the placenta and absence in some of the BEV samples suggesting that BEV DNA may account for most of the reported microbial species and lends support to the belief that the placenta is most often sterile.

The overlap between microbial communities from the placenta and BEV suggests that the microbial signature observed in the placenta can likely come from the BEVs. Our assays and analysis approaches support the reported findings that the placenta does not harbor an actual microbiome (de Goffau et al., 2019; Gschwind et al., 2020; Sterpu et al., 2021; Panzer et al., 2023). Additionally, “Kitome and Flashome” can contribute to false signals as the placental microbiome signal-to-noise ratio is below the threshold of what is perceived as an actual microbiome (Parnell et al., 2017b; Olomu et al., 2020). We postulate that the human placenta harbors BEVs released by microbes residing in the body or entering through the subjects’ air, water, or food. BEVs are normal constituents of human placenta, and they are likely nonimmunogenic particles. Our results provide additional insights into the placental microbiome controversy (Blaser et al., 2021), as DNA amplified from BEVs can be mistaken for the presence of microbes in the placenta.

**Figure 7:**
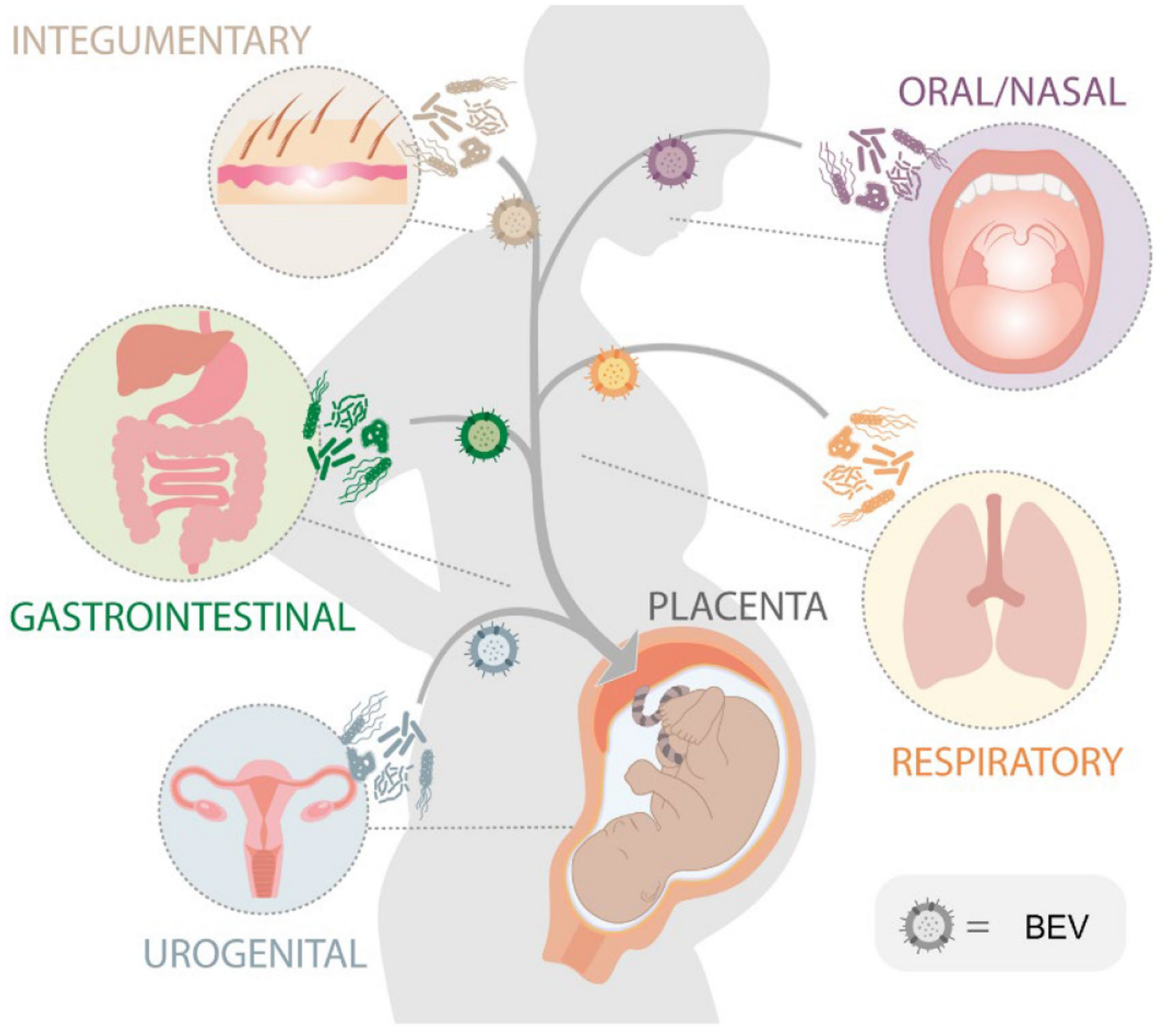
Schematic diagram illustrating the potential sources of bacterial extracellular vesicles (BEVs) that are isolated from the placenta. The hypothesis is that these BEVs, originating from bacteria in various organs within the body, can be released into the bloodstream and subsequently delivered to the placenta during pregnancy.

Placental BEVs are likely deposited as they reach the feto-maternal interface via systemic circulation (Turunen et al., 2023). BEVs in systemic circulation are either from the endogenous (part of the host microbiome) or exogenous sources (consumed via contact or through food; Schaack et al., 2022) (Figure 7). Based on the microbial profile generated in this manuscript, the origin, and the source of BEVs are challenging to predict. Biological specimens like vaginal, skin, fecal, or oral samples were not collected from subjects, and microbiome profiling was not conducted to do a correlative analysis reported previously (Aagaard et al., 2014). It is tempting to postulate that during disease states in other tissues, such as periodontal disease, gastrointestinal infection, and bacterial vaginosis, microbial communities can produce bacterial vesicles that can be transported to the placenta via a hematogenous route or intracellular paracrine way. Bacterial vesicles from pathobionts may result in localized inflammation and pregnancy complications, especially in higher concentrations (Surve et al., 2016). This was previously demonstrated in a mouse model treated with GBS BEVs. Intraamniotic injections of GBS BEVs promoted localized inflammation mimicking features of chorioamnionitis and induced apoptosis in the choriodecidual tissue. It also promoted intrauterine fetal death and preterm delivery (Surve et al., 2016). Besides, BEVs from normally colonized maternal sites (e.g., oral, gut, nasal, skin, mucus lining of organs, and reproductive tract) can reach fetal organs and prime them for self-antigen recognition. This immune recognition may prime the neonatal immune system from developing an inflammatory reaction to normal microbiome exposure. This is clinically relevant as BEVs can be used as potential vaccines during pregnancy.

Bacterial extracellular vesicles can enter placental and maternal decidualized endometrial cells; however, their minimal immunogenicity further supports their natural presence and potential commensalism. However, immunogenicity and PTB induced by *E*. *coli*-derived BEVs support the potential pathogenic effects of BEVs. This hypothesis is supported by a previous study that showed that BEVs derived from pathogenic microbe, Group B *Streptococcus* promoted chorioamnionitis, preterm birth, and fetal demise in a mouse model (Surve et al., 2016). BEVs can propagate and cause placental and maternal uterine tissue inflammation predisposing to preterm birth. Our current study showed that placental BEVs could trigger localized inflammation in immune cells but not in placental and decidual cells; however, this could indicate a nonspecific response to a localized irritation created by the presence of BEVs. We speculate that BEVs and the low dose of antigens they carry, including LPS, may provide a tolerogenic environment and help build the fetal immune repertoire and recognition of self-antigens. Mucosal immunity is expected to develop in the late first trimester, and there is no evidence of microbes in the fetal gut currently. BEVs can contain various antigen repertoire that can come from Gram-negative bacteria and provide immunological programming of the fetus during development to most environmental microorganisms. BEV-based vaccine development has been tested clinically, and many are in use (König et al., 2021; Thapa et al., 2021; Rollier et al., 2022). It is tempting to speculate that BEVs from endogenous sources from the mother may also confer immunological memories to the growing fetus, preparing them for adult life in the environment with these microbes.

Placental specimens used for this study may also determine the quantity and taxa represented by BEVs. Through univariate analysis, we identified *Chryseobacterium*, *Elizabethkingia*, *Enterobacter*, *Citrobacter*, *Janthinobacterium*, *Yersinia*, *Salmonella*, *Serratia*, *Rhodococcus*, *Sphingobacterium*, and *Enterobacter*, as differentially abundant genera between the placenta and BEVs. Except for *Rhodococcus*, these genera are Gram-negative, ubiquitous in nature, coliform bacteria. Most of these bacteria are seen as a part of the normal microbiome in animals; however, some of them may be associated with pathogenicity in humans. The abundance of BEVs DNA typed to environmental bacteria indicates they likely originate from consumed food and drinks followed by absorption into the systemic circulation. *Turicibacter* was the only genus upregulated in the placenta compared to BEVs. However, it was detected in several BEV samples, including within a pair of samples. Additionally, it is essential to consider that among the samples in which *Turicibacter* was detected, the median read count was 59. This prompted us to further include a negative binomial GLM for differential abundance analysis, including placental lab ID as an additional variable. *Yersinia*, *Salmonella*, *Rhodococcus*, and *Turicibacter* were not identified as differentially abundant when factoring in for placental ID at a Bonferroni value of *p* cutoff of 0.05. We conclude that BEVs isolated from the placenta are indicators of a pregnant subject’s natural and environmental exposure rather than actual bacteria colonizing the tissue. The abundance of environmental microbial-derived BEVs and the heterogeneity observed in microbial Taxa suggest interindividual exposure differences and do not necessarily indicate accumulation of BEVs for any specific functions. The placenta gives an interesting perspective as it is a temporary, blood-rich site for filtration where systemically BEVs may accumulate because of the high blood turnover. Additional studies on the blood-rich organs need to be done to study this concept further.

Interestingly, we also detected plant DNA from *Solanum melongena* (eggplant) in our placenta and BEV samples. We hypothesize that these plant DNAs may have come from plant-derived extracellular vesicles (PDEV). PDEVs may be introduced to pregnant patients via environmental exposure or diet. Previous studies showed that PDEV could be derived from numerous edible plants (Wang et al., 2013). They can be obtained from various plant parts, such as the juice, the flesh, or the roots (Urzi et al., 2021; Kim et al., 2022). PDEVs also carry cargo, including proteins, lipids, and nucleic acids (Kameli et al., 2021). Moreover, PDEVs have diverse biological functions, including protection against inflammation and intestinal permeability, anti-cancer property, and promoting host-microbe symbiosis (Rome, 2019). A previous study showed that watermelon-derived EVs decreased apoptosis and increased extravillous trophoblast migration in first trimester placental explants. It also induced the expression of genes involved in syncitialization and increased cell–cell fusion in BeWo cells, ultimately demonstrating the potential impact of PDEVs on placental development, growth, and function (Théry et al., 2018; Timms, 2018). Further studies are needed to determine the potential role of PDEVs in placental function and pregnancy.

This study established a method to isolate BEVs from human tissues and isolated BEVs are successfully characterized. We also showed that BEVs can confound microbiome studies if investigators strictly use nucleic acid-based approaches. Further, microbial isolation and cultures are needed to demonstrate their true existence in the placenta and molecular approaches alone are insufficient to prove their presence. Knowledge of exogenous but inert constituents of the placenta is extremely important clinically as their presence can modify the placental environment locally. This study had several limitations. Our study is limited to normal term Cesarean deliveries, and we have not yet examined pregnancies complicated by infection to see if the microbiome BEV changes. Ongoing studies suggest that BEV from pathogenic bacteria (e.g., *E*. *coli*) can induce preterm birth and this mechanism needs to be explored in detail further. Our study also did not examine the source of BEVs and the contribution of each site to the pool of BEVs observed as this was not one of the objectives of this study. A sampling from the respective study is needed to do a correlative analysis to determine the site of BEV origin and will be one of the future directions. The limitations in the bioinformatics analysis include the following: limited taxonomic resolution due to using a conserved hypervariable region to identify species, biases in PCR and sequencing, and limitations of the reference database because this only identified bacterial species included in the database.

## 5. Conclusion

This study, to our knowledge, is the first study that successfully isolated and characterized BEVs from the placenta. 16S rRNA sequencing revealed that the BEVs isolated from the placenta were from different microbial sources. BEVs and other microbial vesicles from other tissues in the human body can reach the placenta through hematogenous spread from maternal and fetal colonizers, intercellular paracrine pathways, or the placental microbial community. BEVs can confound placental microbiome studies. Our study showed that placental BEVs could trigger localized inflammation in immune cells but not in placental and decidual cells. We propose that the placental BEVs may provide a tolerogenic environment and help develop the fetal immune system to confer protection and robust immune response to bacterial infections in the future.

## Data availability statement

The datasets presented in this study can be found in online repositories. The names of the repository/repositories and accession number(s) can be found below: NCBI—PRJNA872181.

## Ethics statement

Placental specimens used for this study were discarded specimens, and no subject recruitment or consent was required for this study. No subject-related identifiers were collected for data analysis. The University of Texas Medical Branch approved this study as an exempt protocol for human specimens. Written informed consent for participation was not required for this study in accordance with the national legislation and the institutional requirements.

## Author contributions

RM conceived and designed the study and provided funding. OT developed BEV isolation protocols and conducted initial studies, including EM and marker analysis. EG, ER, GB, and RU-G performed placental BEV isolation, DNA extraction, uptake studies, and ELISAs. AK helped with protocols. RP provided microbiome expertise in data interpretation and analysis. JY provided funding support, and KK and GG performed all microbiome assays and analysis. RM, OT, ER, and KK wrote, edited, and reviewed the manuscript. All authors contributed to the article and approved the submitted version.

## Funding

This study is supported by UTMB’s Research Program Development commitment funds to RM. RM is also supported by NIH funds 5R01HD100729-04 and 3UG3TR003283-02S1.

## Conflict of interest

The authors declare that the research was conducted in the absence of any commercial or financial relationships that could be construed as a potential conflict of interest.

## Publisher’s note

All claims expressed in this article are solely those of the authors and do not necessarily represent those of their affiliated organizations, or those of the publisher, the editors and the reviewers. Any product that may be evaluated in this article, or claim that may be made by its manufacturer, is not guaranteed or endorsed by the publisher.
