# Supplementary material for: Amplification of microbial DNA from bacterial extracellular vesicles from human placenta
Source: Front Microbiol. 2023 Jul 13;14:1213234. doi: 10.3389/fmicb.2023.1213234 (PMC10374210; doi:10.3389/fmicb.2023.1213234)
Supplement: Supplementary file 3 [file Data_Sheet_1.docx]

Supplementary Material

Amplification of Microbial DNA from Bacterial Extracellular Vesicles from Human Placenta

Ramkumar Menon, MS, Ph.D^1,*^, Kamil Khanipov, Ph.D^2^, Enkhtuya Radnaa, Ph.D^1^, Esha Ganguly Ph.D^1^, Giovana Fernanda Cosi Bento, MS^1^, Rheanna Urrabaz-Garza, BS^1^, Ananth Kumar Kammala, Ph.D^1^, Jerome Yaklic, MD^1^, Richard Pyles, PhD,^3^ George Golovko, Ph.D^2^, Ourlad Alzeus G. Tantengco, MD, Ph.D^1,4,5,*^

^1^Department of Obstetrics and Gynecology, The Division of Basic Research and Translational Science, The University of Texas Medical Branch at Galveston, TX, USA

^2^Department of Pharmacology and Toxicology, The University of Texas Medical Branch at Galveston, TX, USA

^3^Department of Pediatrics, The University of Texas Medical Branch at Galveston, TX, USA

^4^ Department of Physiology, College of Medicine, University of the Philippines Manila, Philippines

^5^ Department of Biology, College of Science, De La Salle University, Manila, Philippines

*** Correspondence:
Ramkumar Menon, MS, PhD.** Department of Obstetrics and Gynecology, MRB 11.138, 301 University Blvd, The University of Texas Medical Branch, Galveston, TX 77555, Ph 615 335-5564 (cell): 409 772-7596 (lab) E-mail: [ra2menon@utmb.edu](mailto:ra2menon@utmb.edu)

**Ourlad Alzeus G. Tantengco, MD, PhD.** Department of Physiology, College of Medicine, University of the Philippines Manila, Philippines, 1000, E-mail: [ogtantengco@up.edu.ph](mailto:ogtantengco@up.edu.ph)

# Supplementary Data

None.

# Supplementary Figures and Tables

## Supplementary Figures


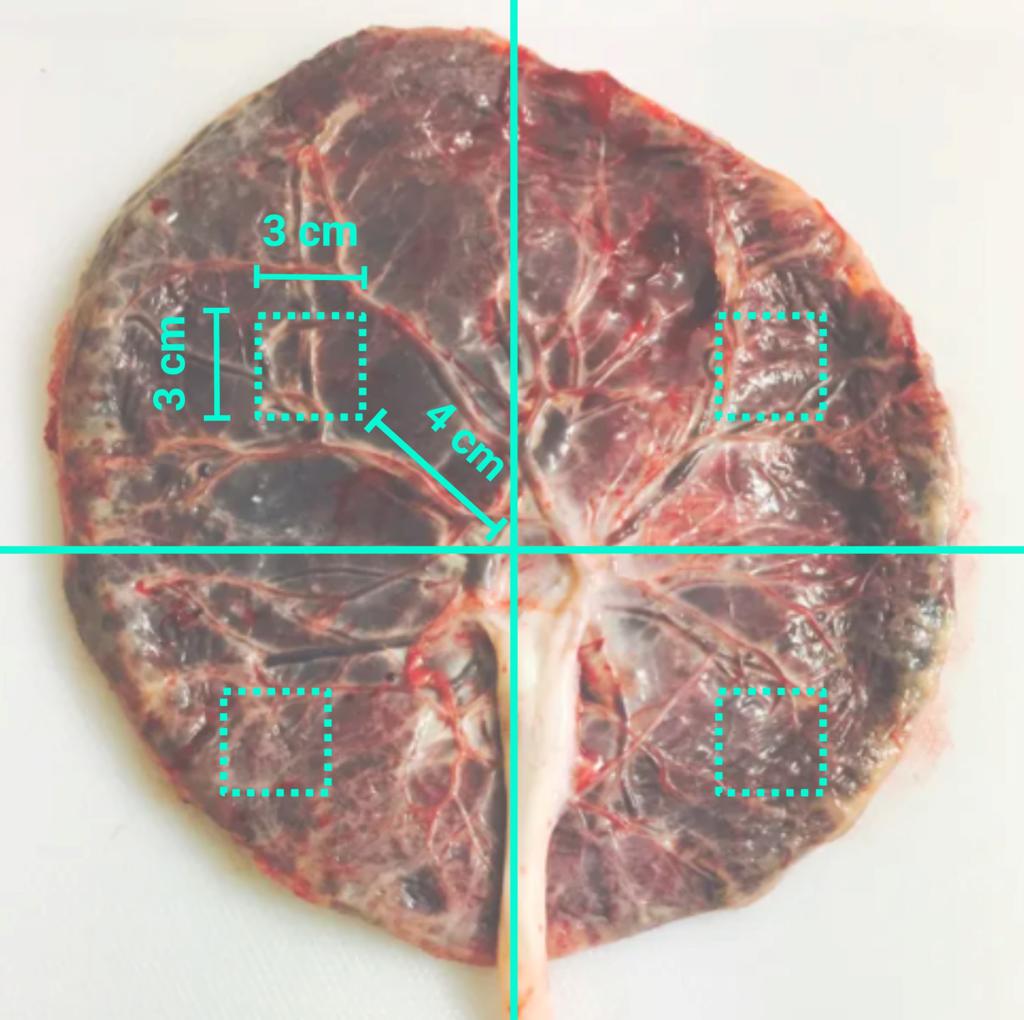


**Supplementary Figure 1. Sites of collection of placental tissues.** Approximately 3×3 cm cross-sectional tissue samples were collected from all four quadrants of the placenta, each at about 4 cm from the cord insertion site. The fetal membrane was not included in the collected placental tissues.


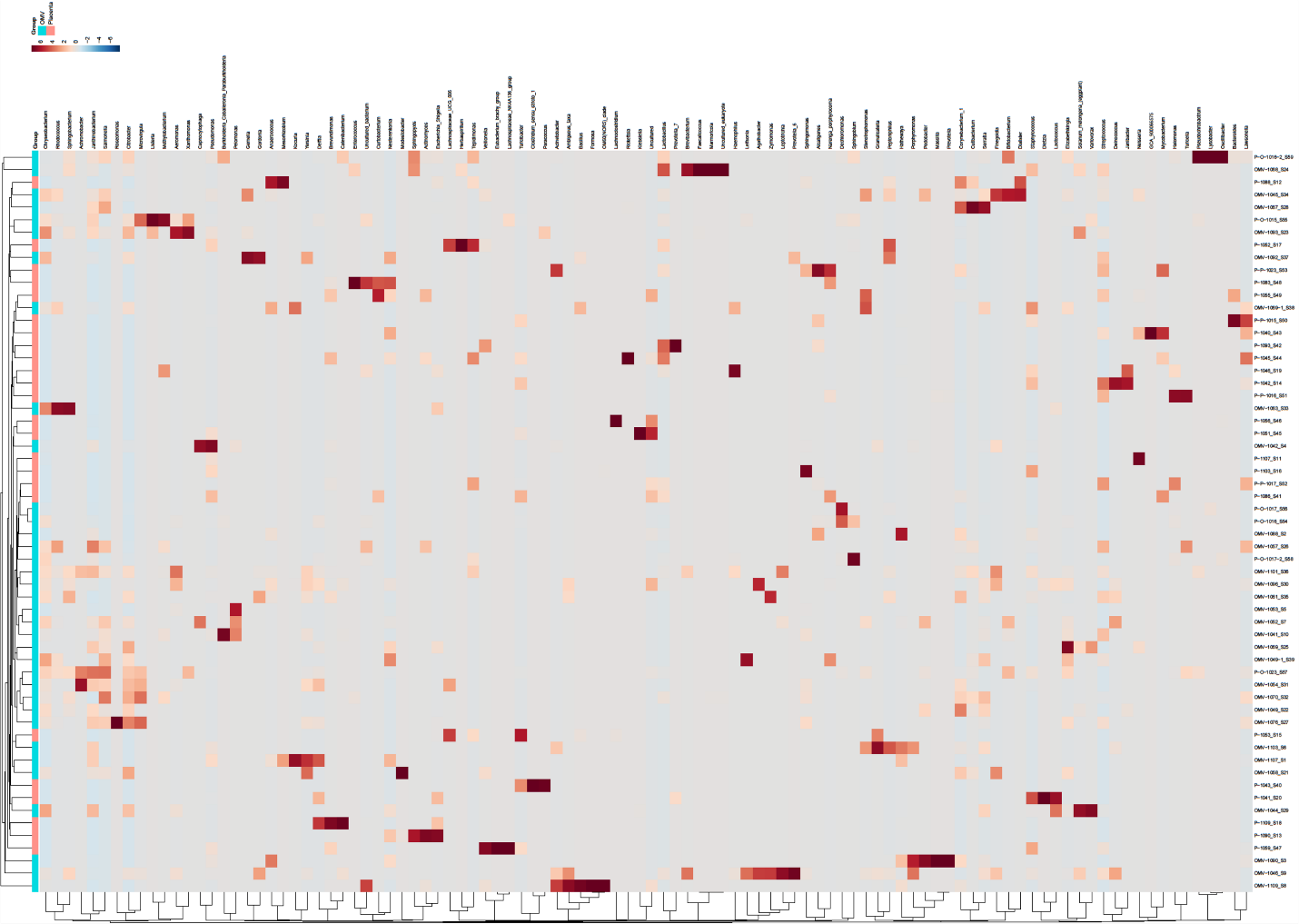
**Supplementary Figure 2.** Heatmap generated using the median ratio (log2) with hierarchical clustering on the genera.


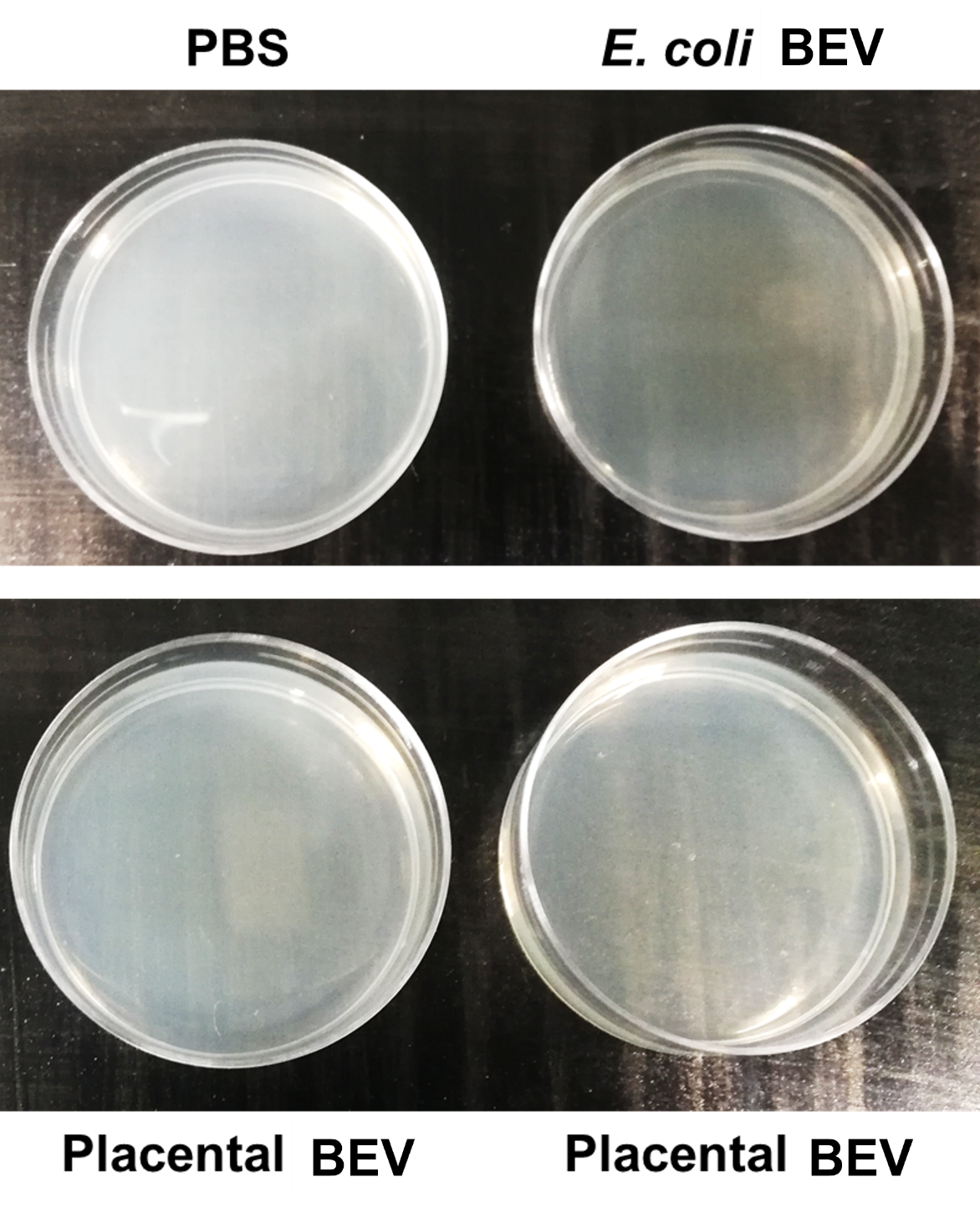


**Supplementary Figure 3.** Representative images of LB-agar plates inoculated with BEV samples from *E. coli* bacterial culture and placental tissues.

**
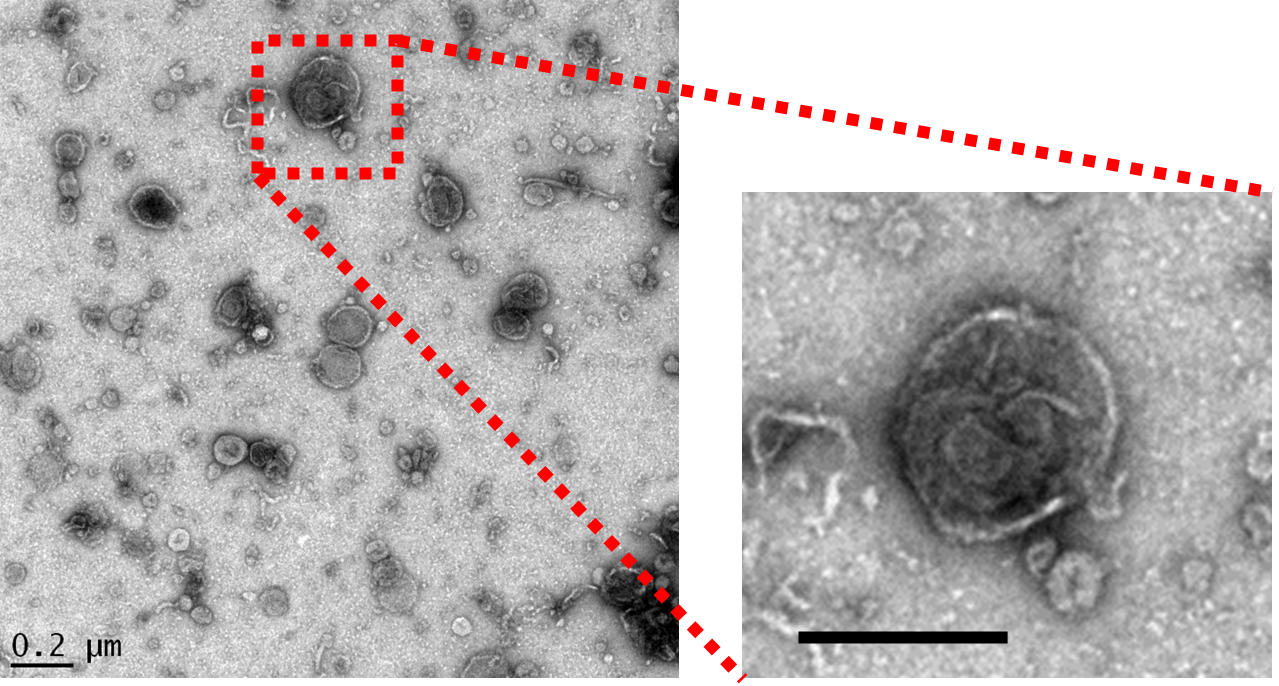
**

**Supplementary Figure 4.** Transmission electron micrograph of bacterial extracellular vesicles isolated from placental tissues show spherical and cup-shaped vesicles. Scale, 200 nm.

**
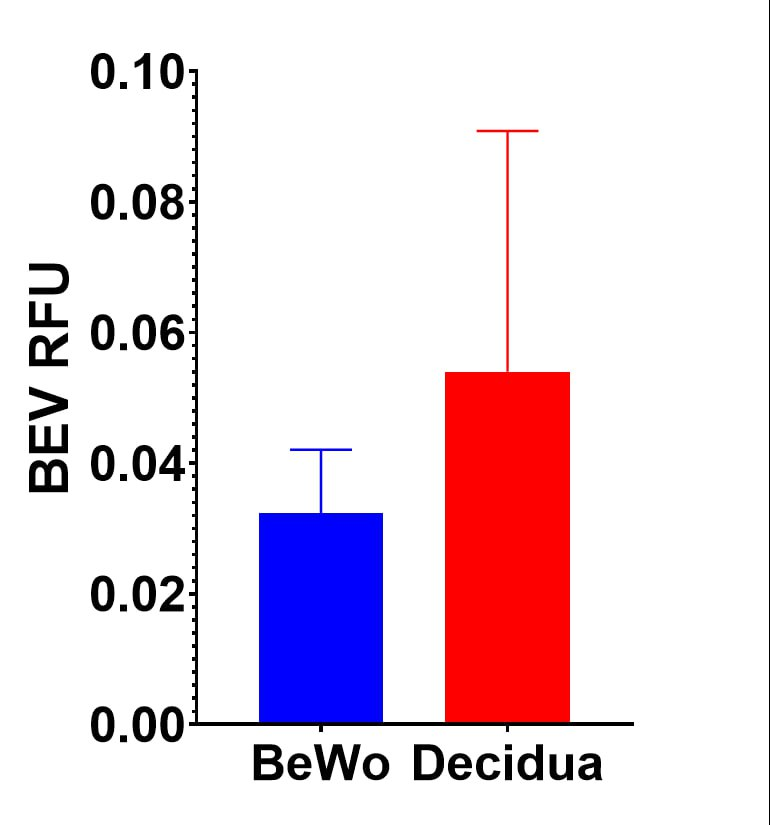
**

**Supplementary Figure 5.** Quantification of placental BEVs taken up the BeWo and hFM-DEC cells. Values are expressed as the mean ratio of vimentin intensity ± SEM. n = 5.


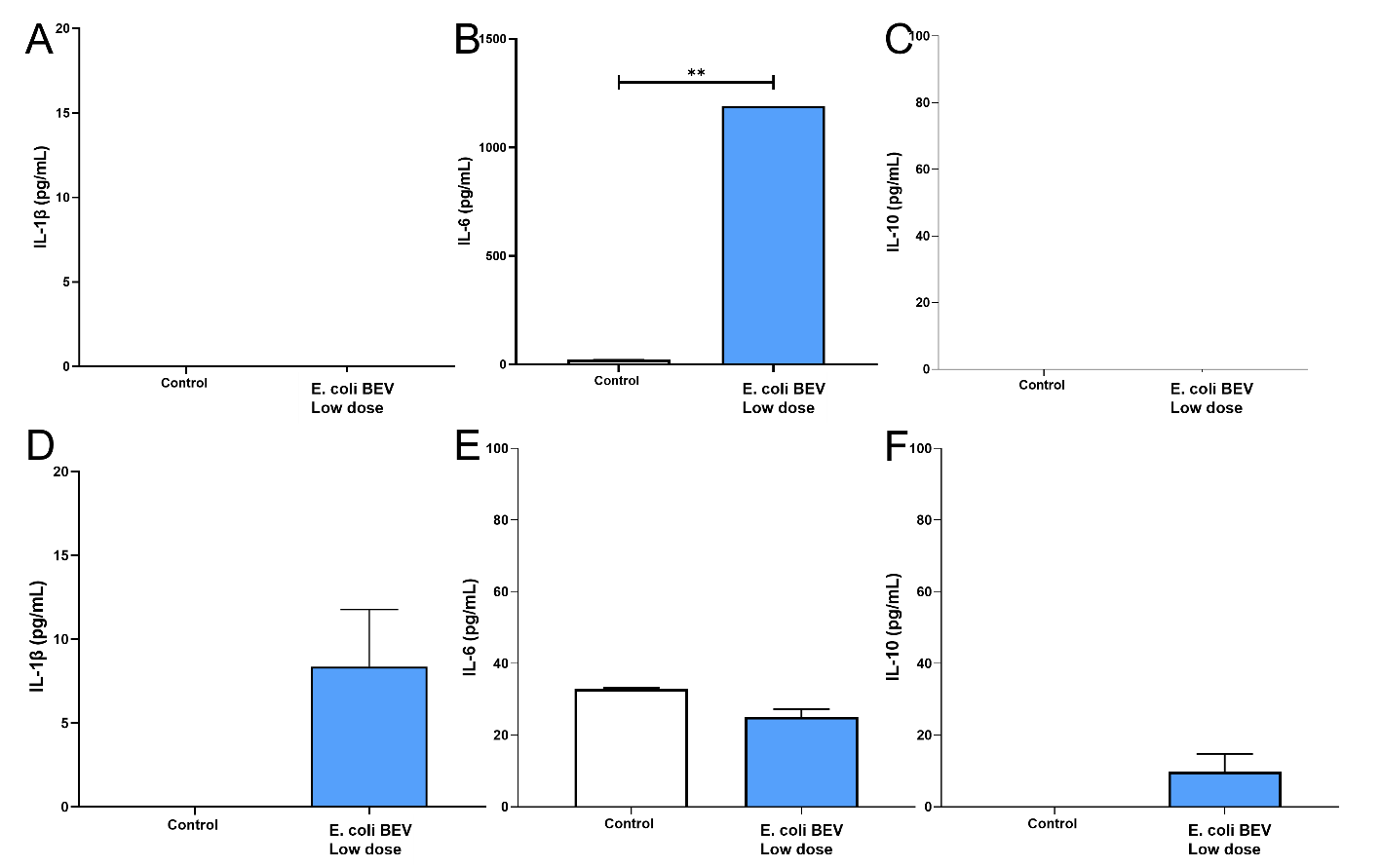
**Supplementary Figure 6.** Cytokine concentrations in maternal hFM-DEC cells **(A-C)** and BeWo cells **(D-F)** after 24 h incubation with *E. coli*-derived BEVs. The concentrations of IL-1β, IL-6, and IL-10 were measured. The values represent mean ± SD (n = 5). **p<0.01.


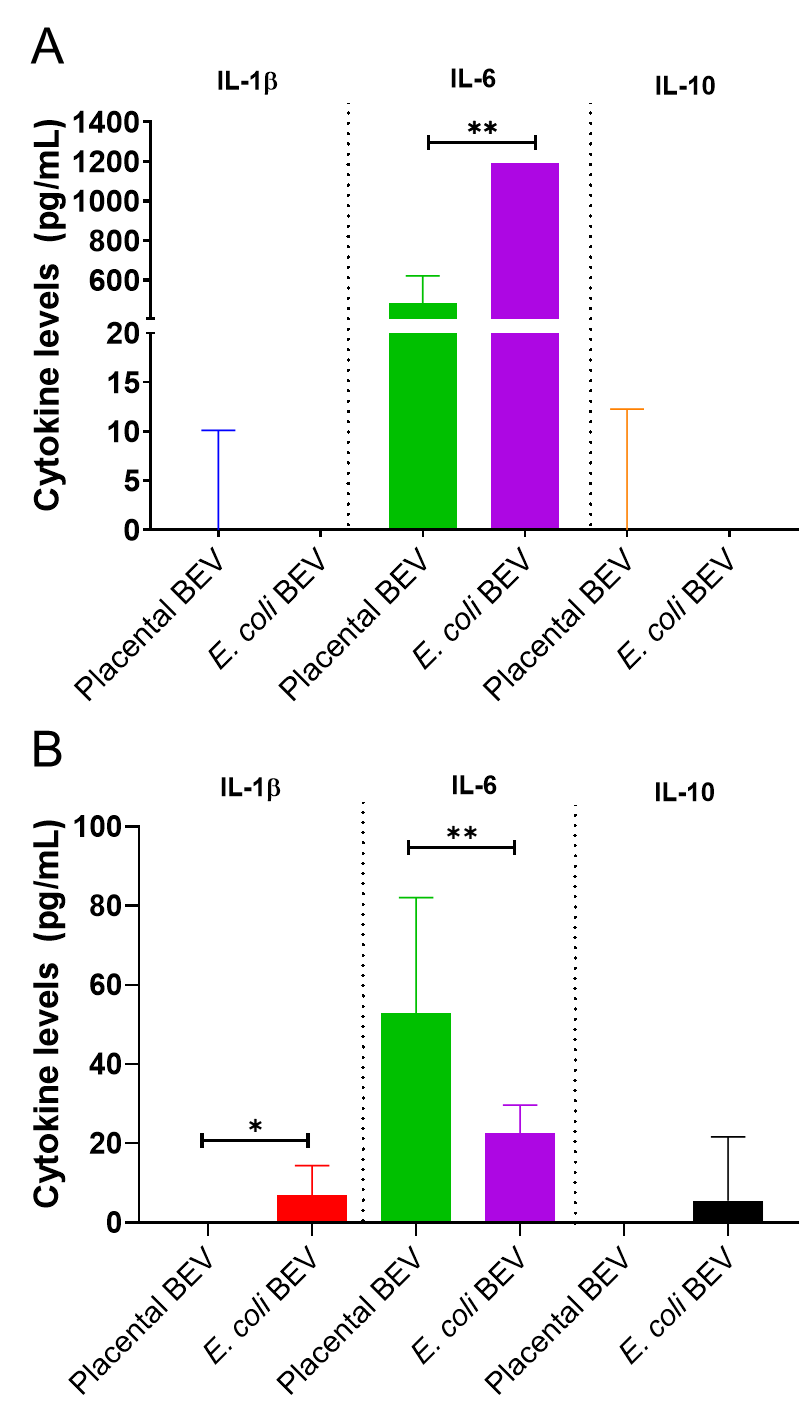


**Supplementary Figure 7.** Cytokine concentrations in maternal hFM-DEC cells **(A)** and BeWo cells **(B)** after 24 h incubation with placenta-derived BEVs vs. *E. coli*-derived BEVs. The concentrations of IL-1β, IL-6, and IL-10 were measured. The values represent media ± interquartile range (n = 5). **p<0.01.
